# Distinct type I and type II toxin-antitoxin modules control *Salmonella* lifestyle inside eukaryotic cells

**DOI:** 10.1038/srep09374

**Published:** 2015-03-20

**Authors:** Damián Lobato-Márquez, Inmaculada Moreno-Córdoba, Virginia Figueroa, Ramón Díaz-Orejas, Francisco García-del Portillo

**Affiliations:** 1 Centro Nacional de Biotecnología-Consejo Superior de Investigaciones Científicas (CNB-CSIC). Darwin, 3. 28049 Madrid. Spain; 2 Centro de Investigaciones Biológicas-CSIC (CIB-CSIC). Ramiro de Maeztu, 9. 28040 Madrid. Spain

## Abstract

Toxin-antitoxin (TA) modules contribute to the generation of non-growing cells in response to stress. These modules abound in bacterial pathogens although the bases for this profusion remain largely unknown. Using the intracellular bacterial pathogen *Salmonella enterica* serovar Typhimurium as a model, here we show that a selected group of TA modules impact bacterial fitness inside eukaryotic cells. We characterized in this pathogen twenty-seven TA modules, including type I and type II TA modules encoding antisense RNA and proteinaceous antitoxins, respectively. Proteomic and gene expression analyses revealed that the pathogen produces numerous toxins of TA modules inside eukaryotic cells. Among these, the toxins Hok_ST_, LdrA_ST_, and TisB_ST_, encoded by type I TA modules and T4_ST_ and VapC2_ST_, encoded by type II TA modules, promote bacterial survival inside fibroblasts. In contrast, only VapC2_ST_ shows that positive effect in bacterial fitness when the pathogen infects epithelial cells. These results illustrate how *S.* Typhimurium uses distinct type I and type II TA modules to regulate its intracellular lifestyle in varied host cell types. This function specialization might explain why the number of TA modules increased in intracellular bacterial pathogens.

Toxin-antitoxin modules (hereafter TA) were discovered in bacteria due to their capacity to stabilize plasmids by interfering with the viability of plasmid-free segregants^1^,^2^,^3^. This phenomenon results from differential stability of the toxin (stable) and the antitoxin (unstable). TA modules are composed of two small genes and classified in five types attending to the antitoxin nature and its mode of action. Antitoxins are either small RNAs (type I and III modules) or proteins (type II, IV and V modules). All known toxins are proteins and exhibit activities ranging from RNAses to DNA gyrase inhibitors^4^,^5^,^6^. TA loci abound in microbial genomes^7^,^8^,^9^,^10^ and are found in archaea^11^ and, in free-living, symbiotic and obligate intracellular bacteria^4^,^5^.

Toxins encoded by TA modules trigger mainly bacteriostatic effects^12^,^13^,^14^. These toxins are implicated in processes as phage abortive infection^15^,^16^; survival in response to nutrient starvation^17^,^18^,^19^,^20^ or to oxidative damage^21^,^22^; biofilm formation^23^,^24^,^25^; and, tolerance to antimicrobial drugs^14^,^26^. Growth arrest caused by TA modules leads to selection of persisters, which are rare slow-growing or dormant cells that normally exist in populations of actively growing cells^14^,^26^. This selection occurs stochastically with no associated heritable genetic alteration.

TA modules have been mostly characterized in bacteria growing in axenic cultures^14^,^26^,^27^. In addition, recent studies implicate TA modules in virulence. Thus, uropathogenic *Escherichia coli* and *Haemophilus influenzae* use type II TA modules to colonize and survive in animal organs^28^,^29^,^30^. *Mycobacterium tuberculosis* up-regulates genes encoding type II TA modules inside macrophages^8^. A type II TA module termed *sehAB*, homolog of the *higBA/relBE* type II TA module, is required for survival of *Salmonella enterica* serovar Typhimurium (*S.* Typhimurium) in mice^31^. Due to the capacity of TA modules to arrest bacterial growth, recent studies have focused in understanding whether TA modules contribute to formation of dormant cells in chronic and persistent infections^32^. Bacterial pathogens that cause these types of infections contain more TA modules than non-pathogenic species that are related phylogenetically^8^,^33^,^34^. Using a macrophage infection model, a recent study reported impaired generation of non-growing *S.* Typhimurium cells in mutants lacking each of the 14 type II TA modules that were tested^32^. An equal contribution of such a large number of TA modules to arrest bacterial growth during infection is, however, intriguing.

*S. enterica* is an intracellular bacterial pathogen associated to persistent infections in humans and livestock^35^. The serovar Typhimurium has been extensively studied in murine models in which the pathogen causes either acute^36^ or chronic infections^37^,^38^. In the animal, *S.* Typhimurium shows limited proliferation inside macrophages^39^. This pathogen also attenuates growth in cultured fibroblasts^40^ and in non-phagocytic cells of the intestinal *lamina propria*^41^. The study reported here includes a comprehensive analysis of *S.* Typhimurium TA modules and shows that a selected group of toxins encoded by these modules might have evolved to control bacterial survival inside host cells. Besides this specialization of functions, our data also implicate for the first time toxins encoded by type I TA modules in promoting pathogen survival in the infected eukaryotic cell.

## Results

### *S.* Typhimurium has a large number of TA modules

**Figure 1:**
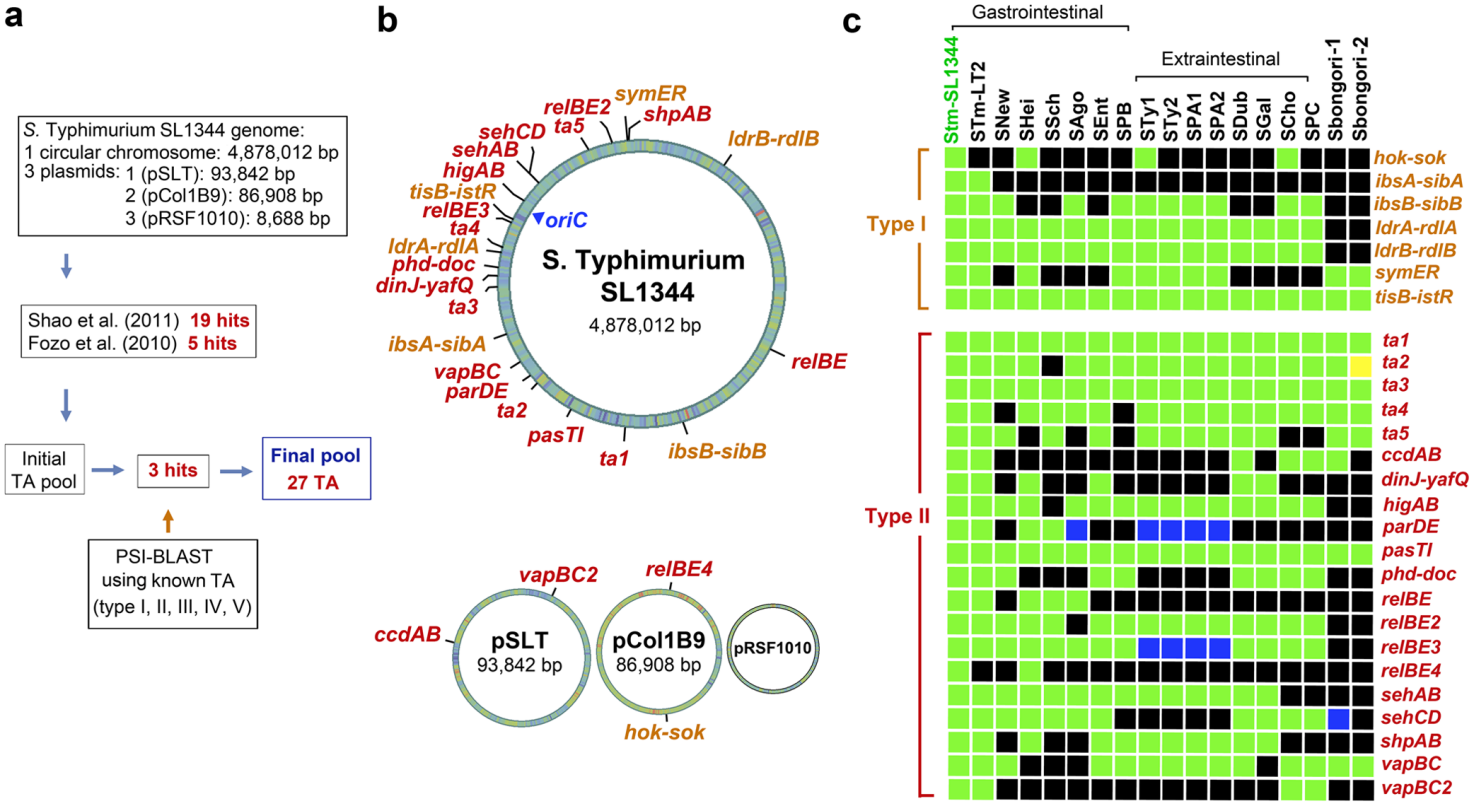
TA modules identified in *S.* Typhimurium strain SL1344. (a) Workflow depicting the *in silico* analyses that led to the identification of 27 putative TA modules in *S.* Typhimurium strain SL1344. The study of Fozo et al.^7^ is focused in type I modules whereas that of Shao et al.^9^ describes the TADB web resource that compiles type II modules from different organisms; (b) distribution of the 27 TA loci identified encoding seven type I (orange) and 20 type II (red) modules in the chromosome and plasmids of strain SL1344; (c) conservation of the 27 TA loci identified in *S.* Typhimurium SL1344 in the genome of other *Salmonella* species and *S.* *enterica* serovars. Homolog search was performed using the tBLASTn tool and as query the protein sequences of toxins and antitoxins identified in *S.* Typhimurium SL1344. The results of this search were contrasted with recent data reported by Nuccio and Baumler^44^, which compared genome sequences of *S. enterica* serovars causing gastrointestinal and extraintestinal pathologies. Abbreviations: STm-SL1344, *S.* Typhimurium SL1344; STm-LT2, *S.* Typhimurium LT2; SNew, *S.* Newport SL254; SHei, *S.* Heildeberg SL476; SSch, *S.* Schwarzengrund CVM19633; SAgo, *S.* Agona SL483; SEnt, *S.* Enteritidis P125109; SPB, *S.* Paratyphi B SP87; STy1; *S.* Typhi CT18; STy2; *S.* Typhi Ty2; SPA1, *S.* Paratyphi A ATCC9150; SPA2, *S.* Paratyphi A AKU_12601; SDub, *S.* Dublin CT_02021853; SGal, *S.* Gallinarum 287/91; SCho, *S.* Choleraesuis SC-B67; SPC, *S.* Paratyphi C RKS4594; *S. bongori*-1, strain N268-08; *S. bongori*-2, strain NCTC12419. Colour code: green, both T and A homologs identified; blue, only T homolog identified; yellow: only A homolog identified; black, no homolog identified. Only hits with e-values ≤ 10^−5^ and pairing encompassing a minimum of 30 amino acids were considered significant.

Our first aim was to identify every putative TA module in the genome of the *S.* Typhimurium virulent strain SL1344 (http://www.ncbi.nlm.nih.gov/genome/152?genome_assembly_id=23044). We used the database described by Fozo et al.^7^ and the web resource TADB (http://bioinfo-mml.sjtu.edu.cn/TADB/)^9^ to search for type I and type II TA modules, respectively. Twenty-four TA loci, accounting for five type I and 19 type II TA modules, were identified (Fig. 1a, Table 1, Supplementary Table S1). Other available tools that predict type II TA modules such as RASTA (http://genoweb1.irisa.fr/duals/RASTA-Bacteria/) did not identify additional hits. PSI-BLAST was also carried out using as queries validated toxins and antitoxins described for TA modules of distinct types (Supplementary Table S2). This PSI-BLAST identified three new putative TA modules, two type I loci (*hok-sok*_ST_ and *symER*_ST_) and one type II locus (*pasTI*_ST_) (Fig. 1a, Table 1, Supplementary Table S1). Recent massive analyses performed for type II^10^ and type III^42^ TA modules were also examined although no new hit was found in the SL1344 genome.

To keep consistently with previous TA nomenclature, we assigned original names to those TA modules displaying high sequence homology to those previously characterized at the functional level in *S.* Typhimurium and other bacteria (e.g. *ccdAB*_ST_, *phd-doc*_ST_, *vapBC*_ST_, *relBE*_ST_, *shpAB*_ST_). Strikingly, most of the genes we identified as encoding putative type I TA modules were not annotated as genes in the *S.* Typhimurium SL1344 genome. This lack of annotation might be related to the marked small size of the toxin and antitoxin genes. Module size ranges from ~50 to ~150 nucleotides in length for the five type I TA modules identified in our study, which are listed in Table 1 with their respective genome coordinates. We also assigned original names to those proteins carrying functional domains of known toxins but not displaying high amino acid sequence similarity with described TA modules. This was the case of proteins encoded by the *relBE3_ST_*, *relBE4_ST_* and *higBA_ST_* loci (Supplementary Table S1). In the case of the type II TA modules named *sehAB*_ST_ and *sehCD*_ST_ by De la Cruz et al.^31^, we maintained that terminology although they are highly homologous to the *higBA/relBE* modules. Lastly, those unknown TA modules for which we found no homologs in the literature or in databases were referred as “TA-(number)-_ST_”, denoting their first identification in *S.* Typhimurium. We also provide the exact genome coordinates for these novel type II TA modules. Tables 1 and Supplementary Table S1 depict the complete list of TA modules identified in our study and their relevant features.

Most of the 27 TA modules predicted in *S.* Typhimurium strain SL1344 have a chromosomal location (Fig. 1b). Four of the 27 TA loci map in two of the three plasmids that the strain SL1344 bears. A *vapBC*_ST_ paralog (here referred as *vapBC2*_ST_) and a *ccdAB*_ST_ homolog map in the pSLT virulence plasmid whereas *hok-sok*_ST_ and *relBE4*_ST_ locate in the pCol1B9 plasmid (Table 1, Fig. 1b). Altogether, these data showed an unsuspected large repertoire of putative TA loci in *S.* Typhimurium, about double of those reported for this pathogen in recent studies^31^,^32^. This large number of TA modules led us to hypothesize about distinct TA modules that could contribute to the fitness of the pathogen inside eukaryotic cells.

### Acquisition of TA modules has been favoured in pathogenic *Salmonella* species

The genus *Salmonella* is composed by the pathogenic species *S. enterica* and the non-pathogenic species *S. bongori*^43^. We reasoned that some of the TA modules, if specialised for contributing to the fitness of intracellular bacteria, might show a narrow distribution restricted to pathogenic bacteria. This specialization of TA modules could also occur among to *S. enterica* serovars associated to gastrointestinal or systemic diseases. To test this, we used as reference the recent study by Nuccio and Baumler^44^, who compared gene content in *S. enterica* serovars that cause either gastrointestinal or systemic pathologies in human and livestock. Those genes encoding TA modules not included in that genome comparative study were used as query in NCBI databases using tBLASTn. Strikingly, up to 17 of the 27 TA loci identified in *S.* Typhimurium SL1344 are absent in the two strains of the non-pathogenic species *S. bongori* with genome sequence available (Fig. 1c). Another prominent feature is the presence of some TA modules in all *S.* *enterica* serovars whereas other TA modules display a narrow distribution in only a few serovars. This is the case of the chromosomal type I TA module *ibsA-sibA*_ST_, restricted to serovar Typhimurium (Fig. 1c); or, *ibsB-sibB*_ST_, which is present in serovars Typhimurium, Newport, Agona, Typhi, Paratyphi A, Paratyphi B, Paratyphi C and Choleraesuis (Fig. 1c). No TA module was found to be restricted to serovars associated to either gastrointestinal or systemic infections (Fig. 1c). Distribution of the plasmid-encoded TA modules *ccdAB*_ST_, *vapBC2*_ST_, *hok-sok*_ST_ and *relBE4*_ST_ was limited to *S.* Typhimurium and other serovars causing system infections such as *S.* Choleraesuis and *S*. Paratyphi C (Fig. 1c). Taken together, these data suggest that acquisition of TA modules might have been favoured in *S. enterica* respect the non-pathogenic species *S. bongori.* Some of these TA modules might also have evolved differently among pathogenic *S. enterica* serovars. This scenario is reminiscent to that reported for other bacterial pathogens as *Mycobacterium tuberculosis*, which has more TA modules than environmental non-pathogenic mycobacteria^34^.

### The TA loci identified in *S.* Typhimurium encode multiple toxins with anti-proliferative activity

**Figure 2:**
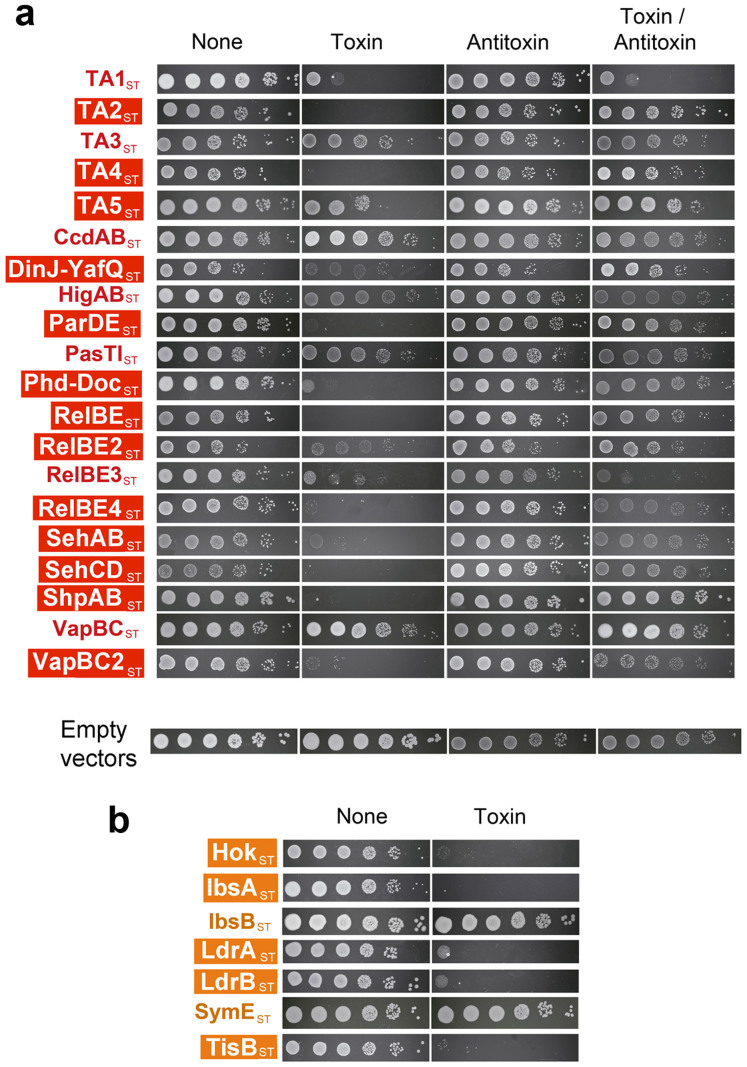
Most toxins and antitoxins predicted in *S.* Typhimurium SL1344 are encoded by *bona fide* functional TA modules. Toxin and antitoxin genes were cloned under P*_lac_* and P*_BAD_* promoters in compatible plasmids. These genes were expressed either independently or combined in the natural host, i.e. *S.* Typhimurium strain SL1344, by plating bacteria onto plates containing IPTG and/or arabinose. Those TA modules highlighted in either red (type II) or orange (type I) boxes correspond to *bone fide* modules. (a) type II modules; (b) type I modules. Assays were repeated in a minimum of three independent experiments. ‘None' means no inducer (IPTG or arabinose) added.

Anti-proliferative and neutralizing activities were next tested for predicted toxins and antitoxins, respectively. Genes encoding toxins and antitoxins of type II TA modules and toxins of type I TA modules were cloned in compatible expression vectors suitable to control expression of each component independently (see Methods). These functional assays were performed in the natural host, *S.* Typhimurium strain SL1344. Thirteen out of the 20 type II TA modules predicted in the genome of strain SL1344 behaved as *bona fide* TA modules (Fig. 2a). Thus, toxins encoded by these thirteen type II TA modules decrease bacterial cultivability, which is restored upon antitoxin co-expression (Fig. 2a). Tests in the putative type I TA modules showed that five of the seven predicted toxins, Hok_ST_, IbsA_ST_, LdrA_ST_, LdrB_ST_, TisB_ST_, impact negatively bacterial growth (Fig. 2b). To our knowledge, these assays are the first demonstrating anti-proliferative activity for some novel toxins predicted here for the fist time and for others previously annotated as putative toxins. These novel and validated toxins include T1_ST_, T2_ST_, T4_ST_, T5_ST_, RelE3_ST_, RelE4_ST_, Hok_ST_, IbsA_ST_, LdrA_ST_, LdrB_ST_ and TisB_ST_ (Table 1, Fig. 2a, 2b). Of interest, some toxins of the type II modules that behave functional in our assays were not as such in previous studies with *S.* Typhimurium grown in liquid culture^31^. This is the case of RelE_ST_, RelE2_ST_, RelE3_ST_, SehAB_ST_, SehCD_ST_, and VapC2_ST_ (Table 1, Fig. 2a). We also found type II TA modules that did not obey the toxicity-neutralization rule, differentiating two groups: i) antitoxins that do not neutralize their cognate toxins, e.g. A1_ST_ and RelB3_ST_ (Fig. 2a); and, ii) toxins that do not have clear effects in cultivability as T3_ST_, HigB_ST_, CcdB_ST_, PasT_ST_, VapC_ST_, IbsB_ST_, and SymE_ST_ (Fig. 2a). Three independent cloning experiments led to identical results. Production of these ‘non-functional' toxins following inducer addition was confirmed by either Commassie staining or Western blotting (Supplementary Fig. S1). A lack of function that brought our attention was that of CcdB_ST_, one of the two type II TA systems that strain SL1344 bears in the virulence pSLT plasmid (Table 1, Fig 1b). When compared to the CcdB of *E. coli* plasmid F, we noticed an R99W amino acid substitution in the *S.* Typhimurium CcdB_ST_ homolog. Residue R99 is reported to be crucial for toxicity in *E. coli*^45^ and an artificial reversion of the R99W mutation in *S.* Typhimurium restores toxin functionality (Supplementary Fig. S2a). Similarly, PasT_ST_ of *S.* Typhimurium shows five out of ten changes in the first ten amino acids when compared to the *E. coli* counterpart. These residues are required for toxin activity^28^. Replacement of this region in PasT_ST_ by the *E.coli* PasT sequence partially restored anti-proliferative activity (Supplementary Fig. S2b). These results demonstrated the presence in *S.* Typhimurium of an unprecedented large number of active toxins encoded by TA modules. In addition, our data indicate that a few TA modules of *S.* Typhimurium might be diverging and loosing some properties respect functional homolog alleles of closely related bacteria.

### *S.* Typhimurium produces inside eukaryotic cells *bona fide* toxins encoded by type II TA modules

**Figure 3:**
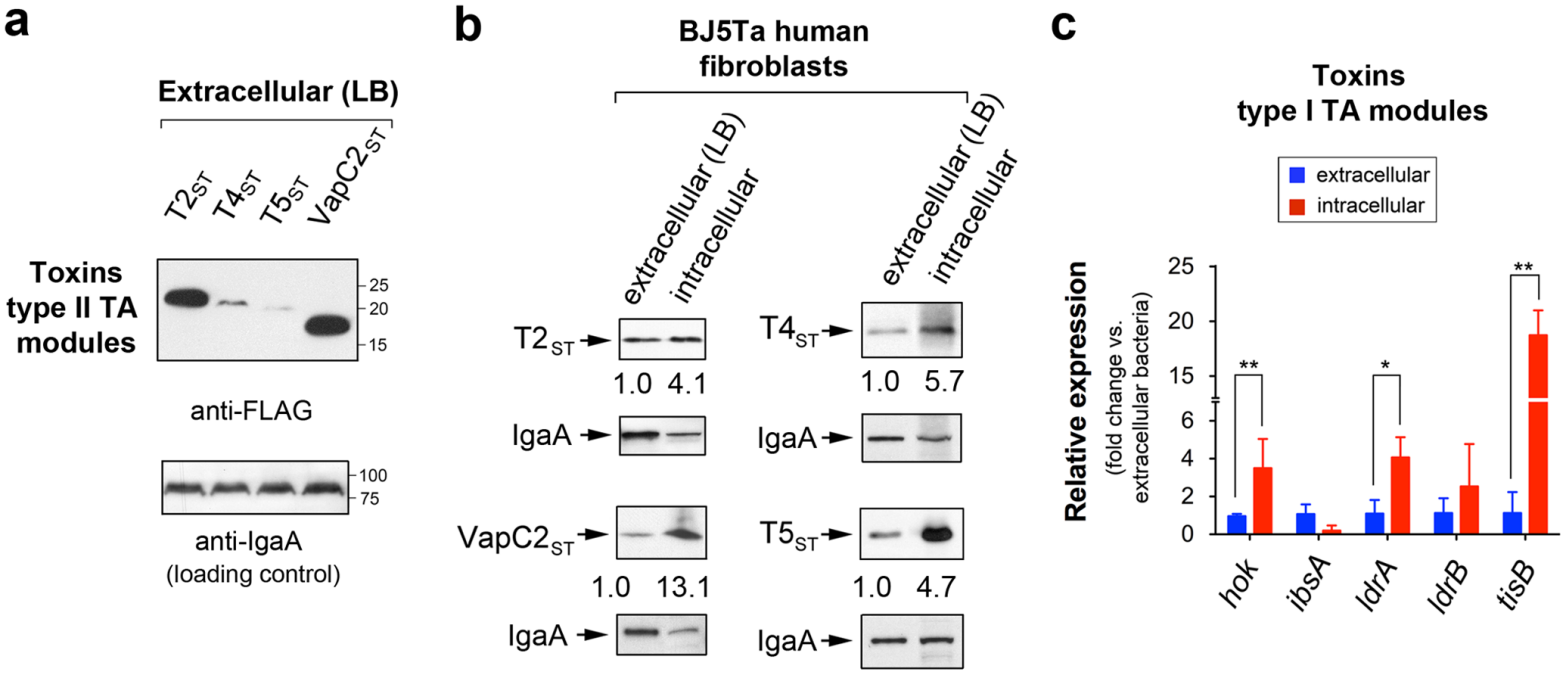
*S.* Typhimurium up-regulates inside fibroblasts *bona fide* toxins encoded by type I and type II TA modules. *S.* Typhimurium was chromosomally tagged with 3xFLAG epitope in genes encoding toxins of type II TA modules detected by proteomics (see Table 2). Protein extracts were prepared from intracellular bacteria at 24 h post-infection of BJ5ta human fibroblasts and extracellular bacteria grown to stationary phase in LB medium. (a) toxins of type II modules detected in extracellular bacteria. Note the distinct relative levels of the toxins examined; (b) synthesis of toxins of type II modules in intracellular bacteria. Note that the four toxins shown (T2_ST_, T4_ST_, T5_ST_, and VapC2_ST_) are produced at higher relative levels inside the eukaryotic cells. The inner membrane IgaA was used as loading control. Numbers below the toxin bands correspond to the relative values determined by densitometry, referred to values measured in extracellular bacteria and corrected by those obtained for the loading control, the IgaA protein; (c) quantitative RT-PCR (qRT-PCR) assays showing the relative expression of five genes encoding toxins of type II modules. Note that three of them displayed significant induction in intracellular bacteria. Data are the means and standard deviations from three independent experiments. *, *P* = 0.01 to 0.05; **, *P* = 0.01 to 0.001, by one-way ANOVA with Dunnett's post-test.

TA modules are in larger numbers in pathogenic versus non-pathogenic *Salmonella* species (Fig. 1). This difference led us to hypothesize that the acquisition of new TA modules could be associated to the emergence of *S. enterica* as an intracellular bacterial pathogen. Given the involvement of TA modules in the generation of growth-arrested cells, we sought to determine whether toxins encoded by these modules control the capacity of *S.* Typhimurium to adapt to distinct intracellular lifestyles within defined host cell types^32^,^46^. Production of components of TA modules by intracellular *S.* Typhimurium was confirmed by highly sensitive gel-free proteomics applied to bacteria directly isolated from human fibroblasts, in which intracellular bacteria show limited proliferation. Despite the small size of toxin and antitoxins (average of ~100 amino acids), high-resolution mass spectrometry identified proteins of some functional type II TA modules in intracellular bacteria. These include the toxins T2_ST_, T4_ST_, T5_ST_, RelE_ST_, Doc_ST_ and VapC2_ST_ and the antitoxins A2_ST_, A5_ST_, ParD_ST_ and VapB2_ST_ (Table 2). The same proteins were also detected by proteomics in samples prepared from extracellular bacteria (Table 2). This result led us to hypothesize whether pathogen adaptation to a lifestyle of limited proliferation inside fibroblasts is modulated by changes in the relative levels of toxins and antitoxins encoded by TA modules. To this aim, we generated strains bearing 3xFLAG tags in the 3′ end of chromosomal and plasmid genes encoding the toxins identified by proteomics. This procedure was not done for the antitoxin-encoding genes as they map upstream of toxin genes and the tagging could result in polar effects. Western blotting assays showed that extracellular bacteria produce rather unequal amounts of toxins T2_ST_, T4_ST_, T5_ST_ and VapC2_ST_ (Fig. 3a). Noteworthy, the level of these four toxins increases by ~ 4- to 13-fold in intracellular bacteria isolated from fibroblasts (Fig. 3b). These values were obtained after correcting by both the amount of the toxin produced by extracellular bacteria and those of the housekeeping membrane protein IgaA detected in extra- and intracellular bacteria (Fig. 3b). The toxin VapC2_ST_ exhibited the highest increase in intracellular bacteria, an observation in concordance to the proteomic data (Table 2). Altogether, these analyses demonstrate that inside eukaryotic cells *S.* Typhimurium induces the expression of functional toxins encoded by *bona fide* type II TA modules.

### *S.* Typhimurium up-regulates inside eukaryotic cells functional toxins encoded by type I TA modules

High-resolution mass spectrometry did not identify toxins of type I modules in protein extracts obtained from either extracellular or intracellular bacteria. These toxins are small peptides of 30–50 amino acids in length, difficult to identify even when using highly sensitive proteomic techniques. To determine if these toxins are also produced by intracellular *S.* Typhimurium, we analysed by quantitative RT-PCR (qRT-PCR) those genes encoding toxins of type I modules that showed anti-proliferative activity: *hok*_ST_, *ibsA*_ST_, *ldrA*_ST_, *ldrB*_ST_, and *tisB*_ST_ (Fig. 2b). Intracellular bacteria isolated from human fibroblasts up-regulate three of these genes, *hok*_ST_, *ldrA*_ST_ and *tisB*_ST_ (Fig. 3c). These data indicate that *S.* Typhimurium up-regulates a selected group of type I TA modules in response to the intracellular environment found in fibroblasts in which bacteria undergo limited proliferation.

### *S.* Typhimurium regulates inside eukaryotic cells the production of toxins encoded by type II TA modules

**Figure 4:**
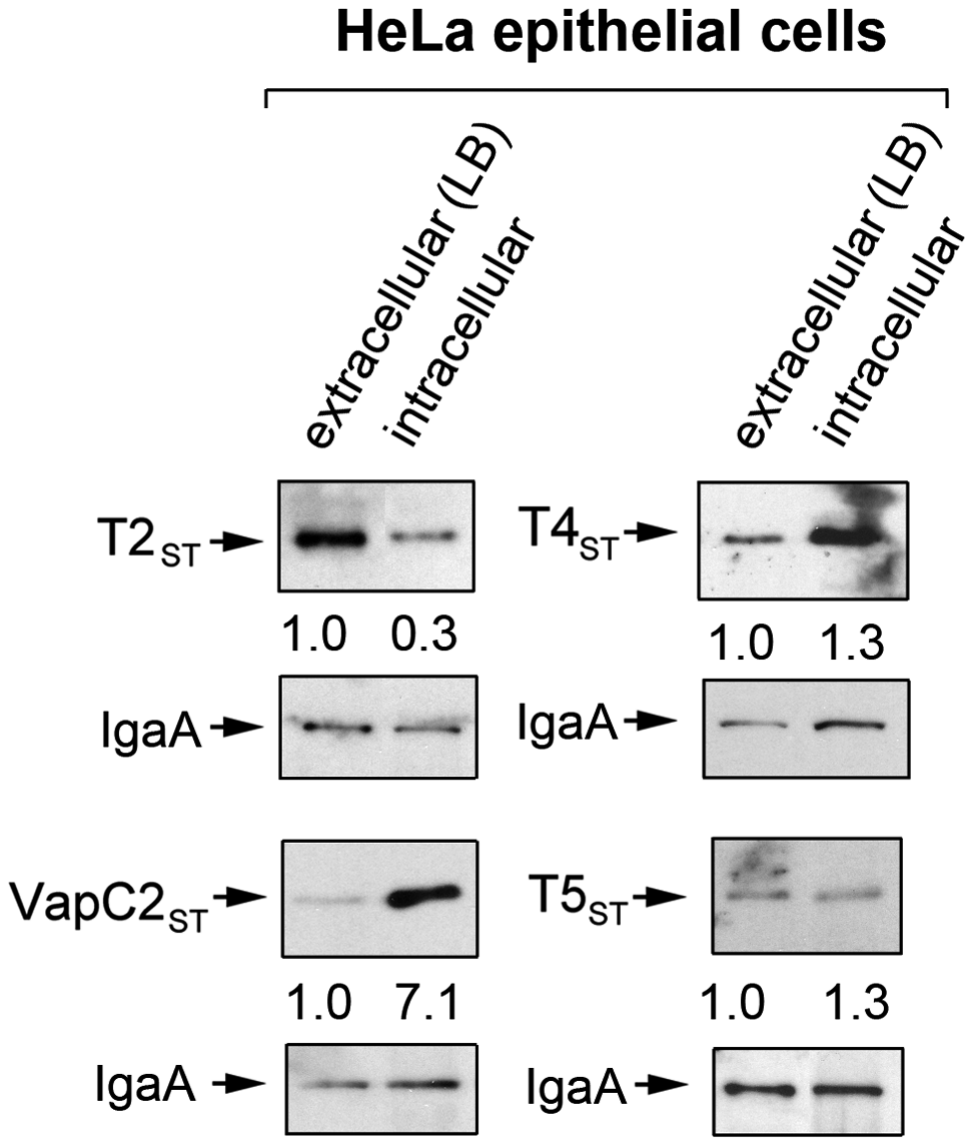
Up-regulation of toxins encoded by type II modules by *S.* Typhimurium is restricted to VapC2_ST_ when growing inside HeLa epithelial cells. 3xFLAG-tagged S. Typhimurium strains were used to infect human HeLa epithelial cells for 16 h. At this time, protein extracts were prepared from intracellular bacteria and tested for toxin protein by Western blotting assay. Numbers below the toxin bands correspond to the relative values determined by densitometry, referred to values measured in extracellular bacteria and corrected by those obtained for the loading control, the IgaA protein. Note that among the four toxins tested only VapC2_ST_ is produced by bacteria at higher relative levels inside the eukaryotic cells. The Western blots shown are representative of a total of three independent experiments.

Next, we reasoned that the high levels of toxins encoded by type II TA modules observed in intracellular bacteria could reflect mechanisms that ensure adaption to an hostile environment that prevents pathogen proliferation. If this was the case, we expected that the relative levels of toxins T2_ST_, T4_ST_, T5_ST_ and VapC2_ST_ could vary if intracellular bacteria were displaying different growth rates. This hypothesis was tested with the 3xFLAG-tagged strains using HeLa epithelial cells, in which *S.* Typhimurium proliferates massively^47^. Quantification of toxin levels in actively growing bacteria isolated from HeLa cells showed that the amount of T4_ST_ and T5_ST_ toxins remain constant compared to extracellular bacteria (Fig. 4). In the case of toxin T2_ST_, its production is however down-regulated by intracellular bacteria (Fig. 4). Of interest, levels of the pSLT plasmid-encoded toxin VapC2_ST_ increase notoriously (~7–14 fold) in intracellular bacteria, irrespective of the capacity of the pathogen to proliferate inside the infected cell (Figs. 3b and 4). These data indicate that *S.* Typhimurium regulates differently the production of some toxins encoded by type II TA modules in varied host cell types.

### Only selected groups of type I and type II TA modules impact *S.* Typhimurium fitness inside eukaryotic cells

**Figure 5:**
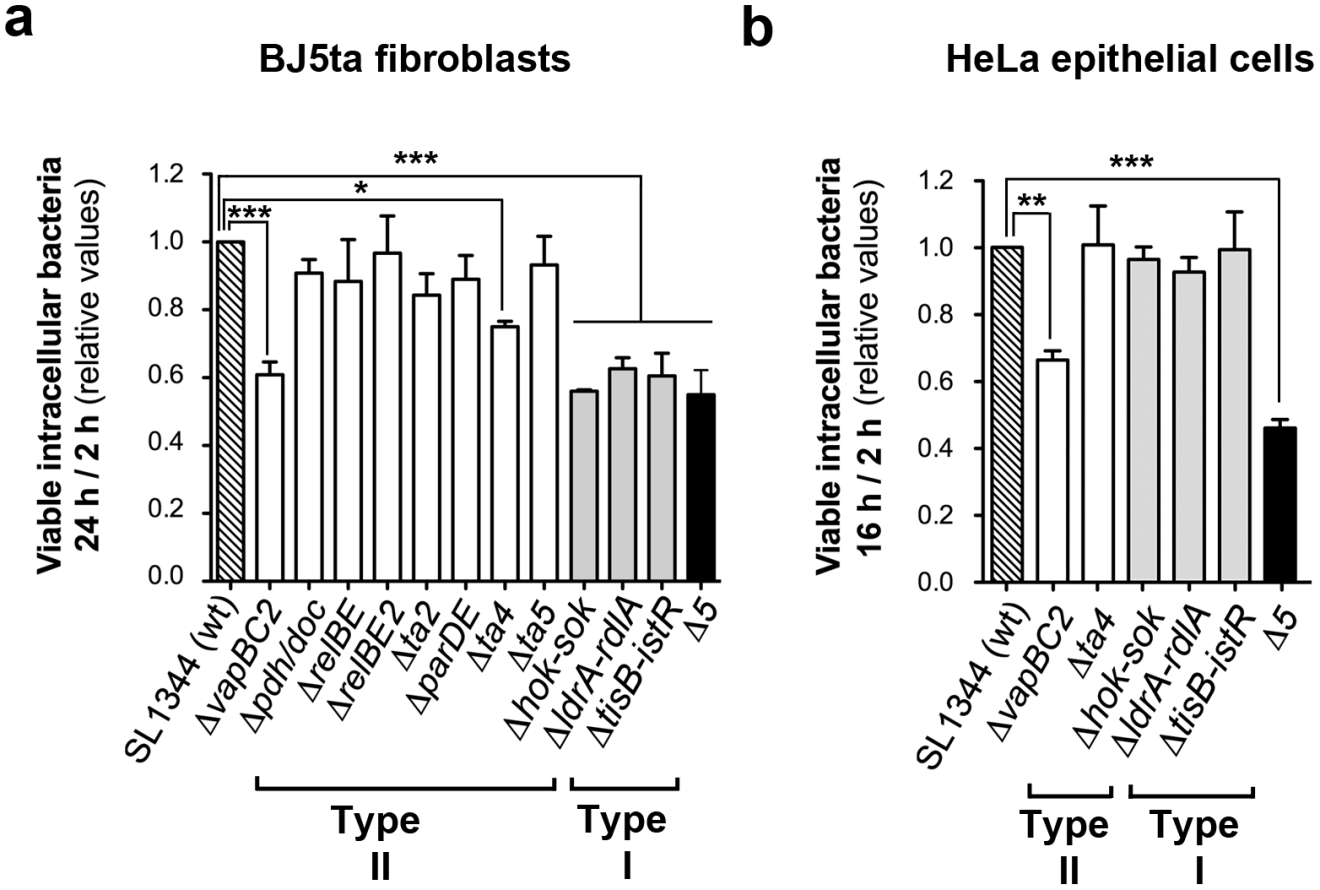
Distinct type I and type II TA modules impact fitness of intracellular *S.* Typhimurium in fibroblasts and epithelial cells. Shown are the survival rates of *S.* Typhimurium mutants lacking the indicated type I (grey bars) and type II (white bars) TA modules. (a) ratio of viable intracellular bacteria at 24 h and 2 h post-infection in human BJ5ta fibroblasts; (b) ratio of viable intracellular bacteria at 16 h and 2 h post-infection in human HeLa epithelial cells. “Δ5” refers to the Δ*hok-sok_ST_* Δ*tisB-istR_ST_* Δ*ldrA-ldrA_ST_* Δ*ta4_ST_* Δ*vapBC2_ST_* mutant. Data are the means and standard deviations from three independent experiments. *, *P* = 0.01 to 0.05; **, *P* = 0.01 to 0.001; ***, *P* = < 0.001, by one-way ANOVA with Dunnett's post-test.

The significance of the enhanced toxin production by *S.* Typhimurium inside fibroblasts compared to epithelial cells was examined with isogenic mutants lacking the respective toxins. We inactivated those *bona fide* TA modules displaying anti-proliferative activity (Fig. 2) and that were induced according to RT-qPCR analyses or detected by proteomics (Table 2, Fig. 3). A total of ten toxin-defective mutants were tested in human fibroblasts, in which *S.* Typhimurium show limited proliferation. Control experiments with these toxin mutants discarded polar effects in the genetic constructions (Supplementary Fig. S3). None of them showed a discernable phenotype in the invasion of the eukaryotic cell lines used (Supplementary Fig. S4) or the growth rate in microbiological media (Supplementary Fig. S5). In contrast, the absence of the three type I TA modules tested, Hok_ST_, TisB_ST_ and LdrA_ST_, or the toxins T4_ST_ and VapC2_ST_, encoded by type II TA modules, impacted negatively the survival of intracellular bacteria inside fibroblasts (Fig. 5a). These toxins were also required for survival of intracellular bacteria in the unrelated rat fibroblast cell line NRK-49F (Supplementary Fig. S6). The Δ*vapC2_ST_* mutant displayed the strongest phenotype with a decrease of up to ~80% in the intracellular survival rate compared to wild-type bacteria (Fig. 5a and Supplementary Fig. S6).

We further tested a multiple mutant lacking the five toxins required for survival of intracellular bacteria (Δ*hok-sok_ST_* Δ*tisB-istR_ST_* Δ*ldrA-ldrA_ST_* Δ*ta4_ST_* Δ*vapBC2_ST_*). This mutant showed a phenotype undistinguishable from individual mutants (Fig. 5a). These data indicate that this set of toxins could be acting in a coordinated manner to prepare bacteria to cope with host defences. Alternatively, some of these toxins might operate in similar if not identical targets or processes, explaining why we did not observe additive effects. Noteworthy, when the same set of mutants was tested in HeLa epithelial cells, only the Δ*vapC2_ST_* mutant and the quintuple mutant showed a clear phenotype. These two mutants proliferate intracellularly with lower growth rates than wild-type bacteria (Fig. 5b). Taken together, these results differentiated toxins of TA modules that impact positively the fitness of intracellular bacteria showing limited proliferation (case of Hok_ST_, TisB_ST_, LdrA_ST_, and T4_ST_) from other toxins such as VapC2_ST_, required for fitness of *S.* Typhimurium inside the host cell irrespective of the growth rate.

## Discussion

The data reported here increase significantly the current list of TA modules known for *S.* Typhimurium and provide experimental evidence for a vast number of *bona fide* TA modules, more than those recently characterized or predicted in databases^9^,^31^,^32^,^48^. We identified 27 TA modules and 20 toxins encoded by these modules have anti-proliferative activity in its natural host, *S.* Typhimurium. This vast repertoire of ‘functional' toxins, which includes members of type I and type II modules, contrasts with the only four functional toxins recognized by De la Cruz et al. based on diminished growth rate when overexpressed in *E. coli*^31^. The proportion of functional toxins characterized here in *S.* Typhimurium is also higher than the reported for *M. tuberculosis*, in which 30 of the 88 predicted and individually-tested TA modules were proved to be functional^8^.

Our study also uncovered apparent ‘non-functional' TA modules. Seven toxin candidates did not affect *S.* Typhimurium growth (T3_ST_, CcdB_ST_, HigB_ST_, PasT_ST_, VapC_ST_, IbsB_ST_ and SymE_ST_). Additionally, two putative type II antitoxins did not neutralize their cognate toxins partners (A1_ST_ and RelB3_ST_). We confirmed that these proteins were efficiently produced from the respective plasmids, so other factors should explain such negative outcome. For CcdB_ST_ and PasT_ST_, which have active orthologs in *E. coli*, site-directed mutagenesis showed that discrete differences in sequence compromise activity in *S.* Typhimurium. This was an unexpected finding that opens new questions regarding the evolution and deterioration (or specialization to other yet unknown functions) of certain TA modules in otherwise rather genetically close bacteria. Lastly, the possibility that a chromosomally-encoded antitoxin could counteract plasmid-encoded toxin cannot be discarded. However, this case seems unlikely given the marked difference in the stability and stoichiometry of the two components. For the ‘inactive antitoxins' A1_ST_ and RelB3_ST_, a possible alternative is that other components are needed for proper antitoxin function, as it was shown for the HigA antitoxin of *M. tuberculosis*^49^. These two proteins could also not correspond to true antitoxins even considering they map in proximity to the toxin gene. A precedent exists in the *mazEF* TA module of *Myxococcus xanthus*, in which antitoxin and toxin genes map apart one from the other^50^.

To our knowledge, the activity of defined TA modules in controlling growth of an intracellular pathogen inside non-phagocytic host cell types was not investigated at the cellular level before this study. The data obtained in fibroblasts uncover a selected class of type I and type II TA modules (Hok_ST_, TisB_ST_, LdrA_ST_, T4_ST_ and VapC2_ST_) that are required for *S.* Typhimurium to survive within this host cell type. Our data provide new information with respect to the recent study of Helaine et al., which analysed type II TA modules in the *S.* Typhimurium-macrophage infection model^32^. First, we demonstrate that toxins encoded by type I TA modules are also as important as those of type II TA modules for the fitness of intracellular *S.* Typhimurium. Second, unlike the work of Helaine et al., which implicated all type II TA modules that they tested (14 modules) in persister cell formation inside macrophages^32^, our data support a clear specialization of functions among distinct type II TA modules. Differences in the host cell type that is invaded by the pathogen might dictate a critical function for distinct sets of TA modules. Thus, among the type II TA modules tested, only the toxins VapC2_ST_ and T4_ST_ impact fitness of *S.* Typhimurium inside fibroblasts. VapC2_ST_ is predicted to have RNAse activity due to the functional domain conservation with its paralog toxin VapC_ST_^51^, which behaved as a ‘non-functional' toxin in our assays (Fig. 2). Regarding T4_ST_, a novel toxin encoded by a TA module discovered in this study, it bears a Gcn5-related acetyl transferase (GNAT) domain. This domain is present in members of a protein superfamily that utilizes acyl coenzyme A (CoA) as donor for the acylation of lysine residues^52^. These enzymes have a large variety of substrates, from nascent endogenous proteins to histones or antibiotics^53^. In *S.* Typhimurium, the action of a GNAT domain-containing protein termed Pat has been related to the control of carbon utilization and metabolic flux via acetylation of several metabolic enzymes^54^. The decreased survival inside fibroblasts experienced by the mutant lacking T4_ST_ supports an important role of metabolic readjustment(s) in ensuring the fitness of intracellular *S.* Typhimurium under conditions of limited growth. This assumption agrees with genome-wide profiling data obtained in this pathogen under identical infection conditions^41^, which showed up-regulation of metabolic genes involved in adaptation to microaerophilic conditions.

TisB_ST_, LdrA_ST_ and Hok_ST_ form the subset of toxins encoded by type I TA modules important for fitness of intracellular *S.* Typhimurium. In *E. coli* these three toxins have been related to dissipation of transmembrane potential and ATP depletion^55^,^56^,^57^. A ‘controlled' action of these toxins in intracellular bacteria could potentially assist *S.* Typhimurium for acquiring the required state of metabolic dormancy in fibroblasts. This model fits with the increased expression of genes encoding Phage-shock proteins (Psp) observed in intracellular *S.* Typhimurium^41^,^58^. The bacterial response involving Psp proteins is triggered as a consequence of stress reducing the energy status of the cell. It is tempting to hypothesize about these Psp proteins as elements balancing the limited damage induced by the toxins encoded by TA modules. This idea could be evaluated in future studies.

Among the five toxins that ensure *S.* Typhimurium fitness inside fibroblasts, LdrA_ST_, Hok_ST_, and VapC2_ST_ are absent in the non-pathogenic species *S. bongori.* Hok_ST_ and VapC2_ST_ are encoded by plasmids that are absent in *S. bongori*, however LdrA_ST_ is encoded by a chromosomal gene. Noteworthy, *ldrA*_ST_ is conserved in all *S. enterica* serovars (Fig. 1), which suggests that acquisition of this particular TA module might be occurred during the evolution and speciation of *S. enterica* as pathogen. Another conclusion of our study involves the different toxins encoded by TA modules that ensure fitness of intracellular *S.* Typhimurium in distinct host cell types. Thus, the behaviour of actively growing *S.* Typhimurium located HeLa epithelial cells is altered exclusively in the absence of the VapC2_ST_ toxin. This is an interesting observation since all toxins tested were shown by Western blotting to be produced by intracellular *S.* Typhimurium in both fibroblasts and epithelial cells, although at varied relative amounts. Based on these findings, not only the type of toxins but also the proportion at which are produced by the pathogen inside the eukaryotic cell might balance the final outcome in terms of persistence or active proliferation. This assumption also considers the importance that the coordination among members of such ‘toxin-cocktail' might have. Lack of a particular toxin in such specialized group could lead to de-regulation of the rest and, as a consequence, to a negative impact on the fitness of intracellular bacteria. This assumption is consistent with two recent studies that demonstrate cross-regulation among distinct TA modules of *E.coli*^59^,^60^.

Intracellular *S.* Typhimurium is exposed within the phagosome to stresses like acidic pH, oxidative and nitrosative reactive molecules and, high osmolarity^61^. This fact could explain the apparent higher content of TA modules in bacterial pathogens that infect eukaryotic cells compared to environmental bacteria. Nonetheless, we did not observe any additive effect following the absence of all those toxins of type I and type II modules that impact positively the fitness of intracellular bacteria. This finding could be in concordance with the model supporting a coordinated action of distinct toxin groups. The loss of this hypothetical coordination due the absence of a particular toxin might not change in quantitative terms when additional toxins are lacking. Precedents for this phenomenon exist in the literature. Thus, the two VapBC TA modules encoded by *H. influenzae* contribute to virulence^29^. However, the phenotype of a double mutant lacking both VapBC modules is indistinguishable from those of the individual mutants.

In summary, the data shown here support a marked specialization of defined TA modules for regulating the intracellular lifestyle of bacterial pathogens. These TA modules would be involved in ensuring pathogen intracellular survival, which is critical for the progression of the infection. It is also worthy to note that, to our knowledge, the data shown here implicate for the first time two plasmid-encoded TA modules, *hok-sok*_ST_ and *vapBC2*_ST_, in infection. Of interest in future studies will be also to characterize the regulators that alter the relative levels of toxins encoded by some of these TA modules when the pathogen resides inside the eukaryotic cell.

## Methods

### Bacterial strains and plasmids used in this study

The *S. enterica* serovar Typhimurium and *E. coli* strains together with plasmids used in this study are listed in Supplementary Table S3.

### Identification of TA modules in *S.* Typhimurium SL1344

Complete nucleotide sequences of the *S.* Typhimurium SL1344 chromosome and the three plasmids carried by this strain were downloaded from NCBI. The respective entries are: NC_016810.1 (chromosome), NC_017720.1 (plasmid 1, pSLT), and NC_017718.1 (plasmid 2, pCol1B9) and NC_017719.1 (plasmid 3, pRSF1010). A total of 27 TA modules were identified (information compiled in Supplementary Table S1). The TADB resource (http://bioinfo-mml.sjtu.edu.cn/TADB/)^9^ and the catalogue published by Fozo et al.^7^, were used to identify type II and type I TA modules, respectively. In the case of the TADB resource, the WU-BLAST 2.0 tool was selected. To upload genome sequences into these resources in an operative manner, the *S.* Typhimurium SL1344 chromosomal sequence was divided in four sections. Three inter-fragment sequences were created to detect possible operons overlapping borders between fragments of consecutive fragments. Plasmids sequences were uploaded un-fragmented. BLOSUM62 was selected as comparison matrix, default parameters for cut-off score and word length, and an e-value of 0.01. To avoid spurious hits, searches were manually cured. Thus, hits were discarded when having more than 650 bp in length, not forming part of an operon (establishing a maximum distance of 150 bp between toxin and antitoxin genes), or previously characterized as genes non-related to TA modules. In a second survey, several already identified toxin and antitoxin sequences (including TA modules of type I, II, III, IV and V) were used as queries to a PSI-BLAST search against genome of *S.* Typhimurium SL1344 (Supplementary Table S2). PSI-BLAST was executed as previously described^62^.

### Cloning of *S.* Typhimurium toxin and antitoxin genes

The expression vectors pACYC184^63^ and pFUS2^64^ were used to clone and express toxin and antitoxin genes with the following modifications. A *P_lac_* promoter was obtained from plasmid pNDM220^65^ as a ZraI/BamHI restriction fragment and introduced in the BamHI site of pACYC184. An optimized Shine-Dalgarno sequence^66^ and a polylinker containing ZraI/SpeI restriction sites (between BamHI and SalI sites), were also introduced. Additionally, *lacIQ* repressor was cloned between PsiI/EcoRV sites to ensure proper transcription control. The same Shine-Dalgarno and polylinker were cloned behind the *P_BAD_* promoter of plasmid pFUS2, between EcoRI and KpnI restriction sites. Candidate toxin and antitoxin genes were cloned in either pACYC184- or pFUS2-based expression vectors. Toxin and antitoxin genes were amplified using Pfu polymerase (Promega) using *S.* Typhimurium genomic DNA as template and primers containing ZraI/SpeI restriction sites (Supplementary Table S4). The fragments were ligated (T4 DNA ligase, Promega) 30 min at room temperature (24°C) and transformed in *S*. Typhimurium SL1344 competent cells. All inserts were sequenced to verify a correct construction of the recombinant plasmid.

### Construction of *S.* Typhimurium mutants defective in TA modules

For disruption of toxin-antitoxin genes, the deletion method described by Maisonneuve et al.^67^, was followed.

### Construction of *S.* Typhimurium recombinant strains expressing 3x-FLAG-tagged toxins

3x-FLAG tagging was performed at the 3′-end of toxin genes in their respective chromosomal or plasmidic locations using the procedure of Uzzau et al.^68^. Oligonucleotide primers used in these procedures are listed in Supplementary Table S4.

### Functional assays to detect toxin and antitoxin activities

Toxicity and neutralization assays were developed based on expression of toxin and antitoxin proteins from the compatible vectors pACYC184 and pFUS2 bearing *P_lac_* or *P_BAD_* promoters, respectively. Induction of the desirable protein was carried out with IPTG (isopropyl β-D-1-thiogalactopyranoside) or arabinose. Most toxins were expressed in pFUS2 and its cognate antitoxins in pACYC184. Corresponding pair components of TA modules were co-expressed in *S*. Typhimurium SL1344. Bacteria were grown overnight in LB medium containing the corresponding antibiotic for each vector and supplemented with 0.4% glucose for those cases in which the toxin gene was cloned in pFUS2. This culture was diluted 1:100 in fresh LB medium and bacteria grown up to an optical density (OD_600_) of 0.3. At this time, serial dilutions made in phosphate buffered saline (PBS) pH 7.0 were plated onto solid media containing toxin and antitoxin inducers (IPTG and/or arabinose). IPTG and arabinose concentrations were adjusted to optimize visualization of toxin and antitoxin activities.

### Distribution of TA modules among *Salmonella* species and serovars

To determine the phylogenetic distribution of the 27 TA modules identified in *S.* Typhimurium SL1344, we first examined the comparative genomic study recently reported by Nuccio and Bäumler^44^. This study analyzed 17 genomes of different *S. enterica* serovars mostly associated to intestinal or extraintestinal infections. In addition, TBLASTN was used: i) to determine the distribution of those TA modules identified in our study that were not listed in the Nuccio and Bäumler compilation; and, ii) to compare the distribution of all 27 identified TA modules in *S.* Typhimurium strain SL1344 with those present two strains of the non-pathogenic species *S. bongori* whose genome sequence is available (strains N268-08 and NCTC12419). For TBLASTN, only hits with e-values of ≤ 10^−5^ with a length of 30 amino acids or over were considered significant.

### Bacterial infection of fibroblasts and epithelial cells

Foreskin human BJ-5ta fibroblasts (ATCC CRL-4001) and HeLa epithelial cells (ATCC CCL-2) were used. These two cell lines were propagated and infected as described^69^. Number of viable intracellular bacteria was determined by plating (in triplicate) serial dilutions of the cell lysate at the different post-infection times. Proliferation index was calculated as the ratio of colony forming units (cfu) counted at 24 h (fibroblasts) or 16 h (epithelial cells) versus 2 h post-infection. Assays were performed in a minimum of three independent repetitions.

### Isolation of intracellular bacteria for proteomic and Western analyses

Protocols optimized for the isolation of intracellular *S.* Typhimurium from infected fibroblasts to obtain bacterial RNA and protein have been described elsewhere^41^,^46^. These large-scale infections were designed with a minimum of 12 BioDish-XL 500-cm^2^ plates (ref. 351040, BD Biosciences) per bacterial strain and post-infection time.

### Toxin/antitoxin expression in intracellular bacteria monitored by Western immunoblot

Isogenic *S.* Typhimurium strains carrying a 3xFLAG tag in defined chromosomal toxin genes were constructed (Supplementary Table S3). These tagged strains were used to infect human BJ-5ta fibroblasts or HeLa epithelial as described^69^. Protein extracts prepared from intracellular bacteria were resolved in SDS-PAGE using 10% polyacrylamide gels and processed for Western blot assays with anti-FLAG antibody (ref. F3165, Sigma-Aldrich). Levels of the *S.* Typhimurium inner membrane protein IgaA were monitored as loading control.

### Proteomic analysis in extra- and intracellular bacteria

Proteome comparison was performed in bacteria grown overnight in LB medium at 37°C and intracellular bacteria collected at 24 h post-infection from human BJ-5ta fibroblasts. Protein extracts were prepared as described^41^,^46^, run in SDS-PAGE using 15% acrylamide gels and stained with ‘Colloidal blue staining kit' (Invitrogen). These extracts were prepared as three biological replicates for intracellular bacteria and two replicates for extracellular bacteria grown to stationary phase. Samples were run by SDS-PAGE, stained and collected as 1-mm width gel slices. These slices were cut in eight pieces, washed in 50 mM ammonium bicarbonate and 50% acetonitrile (ACN) and dehydrated with ACN (100%). Gel pieces were rehydrated in 50 mM ammonium bicarbonate with 12.5 ng/µl trypsin and incubated overnight at 30°C. Tryptic peptides were extracted at 37°C using ACN 100% and 0.5% trifluoroacetic acid (TFA), dried, cleaned using ZipTip (Millipore) and reconstituted in 5 µl 0.1% formic acid/2% ACN. This peptide mix was loaded into a C18-A1 ASY-Column 2 cm precolumn (Thermo Scientific EASY-Column), eluted with a Biosphere C18 column (75 µm inner diameter, 15 cm long, 3 µm particle size) (NanoSeparations), and finally separated using a 150 min gradient with the last 100 min from 2–35% buffer B. Composition of buffer A was: 0.1% formic acid/2% ACN and that of buffer B: 0.1% formic acid in ACN. Flow-rate was 250 nL/min on a nano EASY-nLC (Thermo Scientific) coupled to a nanoelectrospay ion source (Thermo Scientific). Mass spectra were acquired on the LTQ-Orbitrap Velos (Thermo Scientific, San Jose, CA) in the positive ion mode. Full-scan MS spectra (m/z 300-2000) were acquired in the Orbitrap with a target value of 1,000,000 at a resolution of 30,000 at m/z 400. For internal mass calibration, the 445.120025 ion for lock mass was used. Charge state screening was enabled and precursors with charge state unknown or 1 were excluded. After the survey scan, the ten most intense precursor ions were selected for CID-HCD MS/MS fragmentation. Peptide identification was performed in both CID (Collision Induced Dissociation) and HCD (Higher-Energy Collision Induced Dissociation) spectra. For CID fragmentation the target value was set to 10,000 and normalized collision energy to 35%. For HCD, target value was set to 50,000 and collision energy was set to 45%. Dynamic exclusion was applied during 30 s. MS data were analyzed with Proteome Discoverer (version 1.3.0.339) (Thermo Fisher, San Jose, CA) using standardized workflows with Sequest search engine. MS/MS spectra were searched against an in-house created FASTA database with the sequences of the toxin and antitoxin protein identified *in silico* in *S.* Typhimurium strain SL1344 (Table 1, Supplementary Table S1). Search parameters included a maximum of two missed cleavages allowed, carbamidomethylation of cysteines as a fixed modification, and oxidation of methionine as variable modifications. Precursor and fragment mass tolerance were set to 10 ppm and 0.8 Da, respectively. Identified peptides were filtered by false discovery rate of 0.01 using Percolator.

### Real-time quantitative PCR

Large scale infection of human BJ-5ta fibroblasts was performed as described^41^. Total RNA (prokaryotic and eukaryotic RNA) was isolated by addition of 1 ml of Trizol reagent (Invitrogen) following indications of manufacturer and co-precipitated with 20 μg of glycogen (Roche). RNA samples were treated with DNAseI for 1 hour at 37°C (Turbo DNA-free kit Ambion/Applied Biosystems). cDNA libraries were constructed from 1 or 2 μg of RNA obtained from extracellular or intracellular bacteria, respectively (High-capacity cDNA archive kit, Applied Biosystems). For qPCR, the Power Sybr Green PCR master mix (Applied Biosystems) was used in a 10 μl final volume and reactions were carried out in an ABI Prism 7500 equipment following standard reaction conditions according to manufacturer's recommendations. Each cDNA sample was run in triplicate and expression data from each condition were obtained from three independent experiments.

### Statistical analyses and densitometry

Statistical significance was analyzed with GraphPad Prism v5.0b software (GraphPad Inc.,) using one-way analysis of variance (ANOVA) with Dunnett's multiple comparison post-test. A *P* value ≤ 0.05 was considered significant. Densitometry on toxins bands obtained in the Western blotting assays was performed using ImageJ, made available to the public by National Institute of Health, USA.

## Author Contributions

D.L.-M., R.D.-O. and F.G.-d.P. conceived and designed the experiments for this study. D.L.-M., I.M.C. and V.F. performed the experiments. F.G.-d.P. wrote the manuscript. All authors discussed the data and made comments on the manuscript.

## Supplementary Material

Supplementary Information Supplementary Information

## Figures and Tables

**Table 1:** TA loci identified in the genome of *S.* Typhimurium strain SL1344

TA locus (*)	TA module type	Coordinates/gene ID (†)	Identification method/Reference
*hok-sok*_ST_ (‡)	I	41921–42073	PSI-BLAST
*ldrB-rdlB*_ST_	I	466721–466936	7
*ibsB-sibB*_ST_	I	2211602–2211658	7
*ibsA-sibA*_ST_	I	3383044–3383103	7
*ldrA-rdlA*_ST_	I	3829510–3829724	7
*tisB-istR*_ST_	I	4019333–4019842	7
*symER*_ST_	I	SL4454 (*symE*_ST_)	PSI-BLAST
*relBE*_ST_	II	SL1480 – SL1479	9
*ta1*_ST_	II	SL2380 - SL2379	9
*pasTI*_ST_	II	SL2659 - SL2658	PSI-BLAST
*ta2*_ST_	II	SL2885 - SL2884	9
*parDE*_ST_	II	SL2935 - SL2936	9
*vapBC*_ST_	II	SL3012 - SL3011	9
*ta3*_ST_	II	SL3437 - SL3438	9
*dinJ-yafQ*_ST_	II	SL3484 - SL3483	9
*phd-doc*_ST_	II	SL3525 - SL3524	9
*ta4*_ST_	II	SL3618 - SL3617	9
*relBE3*_ST_	II	SL3744 - SL3743	9
*higBA*_ST_	II	SL3866 - SL3867	9
*sehAB*_ST_	II	SL3976 - SL3977	9
*sehCD*_ST_	II	SL3979 - SL3980	9
*ta5*_ST_	II	SL4254 - SL4253	9
*relBE2*_ST_	II	SL4379 - SL4380	9
*shpAB*_ST_	II	SL4459 - SL4460	9
*ccdAB*_ST_ (‡)	II	PSLT027 - PSLT028	9
*vapBC2*_ST_ (‡)	II	PSLT107 - PSLT106	9
*relBE4*_ST_ (‡)	II	SLP2_0004 - SLP2_0003	9

(*) Subscript ST is added for clarification purposes to refer exclusively to TA modules characterized functionally in *S.* Typhimurium in this study.

(†) Coordinates and gene ID of *S.* Typhimurium strain SL1344 based on annotations and sequences deposited in NCBI with entries NC_016810.1 (chromosome), NC_017720.1 (plasmid 1, pSLT), and NC_017718.1 (plasmid 2, pCol1B9). Toxin-encoding genes are underlined.

(‡) TA loci mapping in plasmids.

**Table 2:** Toxins and antitoxins of *S.* Typhimurium TA modules detected by mass spectrometry in bacteria isolated from fibroblasts and in bacteria grown in LB medium

			Extracellular (†)			Intracellular (‡)		
Accesion Number	Protein (*)	Mass (Da)	Unique peptides	PSMs	Coverage (%)	Unique peptides	PSMs	Coverage (%)
SL1479	RelE_ST_	11,070	3	8	29.79	1	4	9.57
SL1480	RelB_ST_	9,086	1	2	23.17	-	-	-
SL2379	A1_ST_	13,536	4	19	33.33	-	-	-
SL2380	T1_ST_	14,966	4	6	34.62	-	-	-
SL2658	PasI_ST_	10,769	3	6	31.25	-	-	-
SL2659	PasT_ST_	17,725	2	3	13.29	-	-	-
SL2884	A2_ST_	10,673	2	5	28.13	2	2	31.25
SL2885	T2_ST_	19,059	2	4	13.14	2	4	20.00
SL2936	ParD_ST_	10,369	-	-	-	1	1	7.69
SL3011	VapC_ST_	14,930	-	-	-	1	1	21.21
SL3012	VapB_ST_	14,276	2	3	13.60	1	2	6.40
SL3437	T3_ST_	22,767	11	27	68.00	8	27	47.00
SL3484	DinJ_ST_	9,442	2	4	24.42	-	-	-
SL3524	Doc_ST_	13,580	1	2	7.38	1	2	10.66
SL3617	T4_ST_	17,693	2	5	22.36	1	3	8.07
SL3744	RelB3_ST_	11,754	4	6	36.45	-	-	-
SL3976	SehB_ST_	15,915	1	2	7.75	-	-	-
SL3979	SehC_ST_	12,326	1	1	8.33	-	-	-
SL4253	A5_ST_	10,972	1	2	9.28	2	4	23.71
SL4254	T5_ST_	17,636	1	1	7.98	1	6	7.98
SL4460	ShpB_ST_	11,368	2	3	39.00	-	-	-
SL4459	ShpA_ST_	11,289	2	2	22.11	-	-	-
SLP2_0003	RelB4	10,176	1	1	7.87	-	-	-
SLP2_0004	RelE4_ST_	10,888	1	4	15.05	-	-	-
PSLT028	CcdB_ST_	11,581	3	13	40.59	3	8	40.59
PSLT107	VapB2_ST_	8,636	1	2	10.53	2	3	22.37
PSLT106	VapC2_ST_	14,879	2	6	21.21	3	7	29.55

(*) Subscript ST is added for clarification purposes to refer exclusively to TA protein characterized functionally in *S.* Typhimurium (see Fig. 2). Toxins are underlined.

(†) Protein extracts prepared from bacteria grown in LB medium to stationary phase at 37°C in non-shaking conditions. Two independent experiments were performed. PSM, peptide spectrum match.

(‡) Protein extracts were prepared from non-growing intracellular bacteria at 24 h post-infection of BJ5ta human fibroblasts. Three independent experiments were performed. PSM, peptide spectrum match.
