# Supplementary material for: Distinct type I and type II toxin-antitoxin modules control Salmonella lifestyle inside eukaryotic cells
Source: Sci Rep. 2015 Mar 20;5:9374. doi: 10.1038/srep09374 (PMC4366850; doi:10.1038/srep09374)
Supplement: Supplementary Information [file srep09374-s1.pdf]

SUPPLEMENTARY INFORMATION

Distinct type I and type II toxin-antitoxin modules control *Salmonella* lifestyle inside eukaryotic cells

Damián Lobato-Márquez, Inmaculada Moreno-Córdoba, Virginia Figueroa, Ramón Díaz-Orejas & Francisco García-del Portillo

Supplementary Table S1. Compiled information of the 27 TA modules characterized in *S.* Typhimurium strain SL1344.

Supplementary Table S2. Components of known TA modules used as PSI-BLAST queries.

Supplementary Fig. S1: Production of toxins and antitoxins encoded by type I and type II TA modules exhibiting no activity in functional assays.

Supplementary Fig. S2: Recovery by site directed mutagenesis of anti-proliferative activity in the non-functional toxins $CCdB_{ST}$ and $PasT_{ST}$ of *S.* Typhimurium strain SL1344.

Supplementary Fig. S3: Control experiments discard polar effects due to genetic procedure involving replacement of functional TA loci.

Supplementary Fig. S4: Invasion rate of two fibroblast cell lines (BJ5ta and NRK-49F) and an epithelial cell line (HeLa) shown by *S.* Typhimurium strains lacking *bona fide* type I and type II TA modules.

Supplementary Fig. S5: Growth curves in LB medium of defined *S.* Typhimurium mutants lacking type I and type II TA modules required for survival inside eukaryotic cells.

Supplementary Fig. S6: Survival of defined *S.* Typhimurium mutants lacking type I and type II TA modules in NRK-49F rat fibroblasts.

Supplementary Table S3. Bacterial strains and plasmids used in this study.

Supplementary Table S4. Oligonucleotides used as primers in this study.

Supplementary Table S1. Compiled information of the 27 TA components identified in *S.* Typhimurium strain SL1344, which includes gene orientation, functional domains of toxins and antitoxins and ortholog genes in strain LT2.

Toxin-Antitoxin domain*	Family†	Toxin-Antitoxin gene §	Gene ID strain SL1344	Gene ID strain LT2	Coordinates genome *S.*Typhimurium SL1344	Orientation (strand)
Xre-like domain	-	*a1*	SL2379	STM2413	2527069- 2527825	Coding
Xre-like domain		*t1*	SL2380	STM2414		
RHH-like domain	-	*a2*	SL2884	STM2904	3069908- 3070722	Coding
GNAT-like domain		*t2*	SL2885	STM2905		
RHH-like domain	*parDE*	*parD*	SL2936	STM2955.S	3124178- 3123641	Complementary
RelE-like domain		*parE*	SL2935	STM2954.1n		
YhfG -like domain	-	*a3*	SL3438	STM3471	3642596- 3641837	Complementary
Fic-like domain		*t3*	SL3437	STM3470		
RHH-like domain	-	*a4*	SL3618	STM3652	3859811- 3859051	Complementary
GNAT-like domain		*t4*	SL3617	STM3651		
Xre-like domain	*relBE*	*relB3*	SL3744	STM3778	4000107- 4000773	Coding
RelE-like domain		*relE3*	SL3743	STM3777		
Xre-like domain	(*higBA)*	*sehB*	SL3976	STM4030.S	4261841- 4261087	Complementary
RelE-like domain	*relBE*	*sehA*	SL3977	STM4031		
Xre-like domain	(*higBA)*	*sehC*	SL3979	STM4032.2N	4263074- 4263676	Coding
RelE-like domain	*relBE*	*sehD*	SL3980	STM4033		
RHH-like domain	-	*a5*	SL4253	STM4317	4583140- 4583921	Coding
GNAT-like domain		*t5*	SL4254	STM4318		
RHH-like domain	-	*shpB*	SL4460	STM4529	4806232- 4806808	Coding
COG2929-like domain		*shpA*	SL4459	STM4528		
RHH-like domain	*relBE*	*relB4*	SLP2_0003	SLP2_0003	2395- 2942	Complementary
RelE-like domain		*relE4*	SLP2_0004	SLP2_0004		
MazF-like domain	*ccd*	*ccdB*	PSLT028	PSLT028	73746- 73221	Complementary
RHH-like domain		*ccdA*	PSLT027	PSLT027		
RHH-like domain	*relBE*	*dinJ*	SL3484	STM3517	3701753- 3701215	Complementary
RelE-like domain		*yafQ*	SL3483	STM3516		

Toxin-Antitoxin domain*	Family†	Toxin-Antitoxin gene	Gene ID strain SL1344	Gene ID strain LT2	Coordinates genome *S.*Typhimurium SL1344	Orientation (strand)
Phd-like domain	*phd-*	*phd*	SL3525	STM3559	3749850- 3749258	Complementary
Fic-like domain	*doc*	*doc*	SL3524	STM3558		
COG5606 -like antitoxin domain	(*higBA*) *relBE*	*higA*	SL3866	STM3906	4137213- 4137868	Coding
RelE-like domain		*higB*	SL3867	STM3907		
-		*pasI*	SL2658	STM2686	2837522- 2836766	Complementary
-		*pasT*	SL2659	STM2687		
Phd-like domain	*relBE*	*relB*	SL1480	STM1551	1583940- 1583418	Complementary
RelE-like domain		*relE*	SL1479	STM1550		
RHH-like domain	*relBE*	*relB2*	SL4379	STM4449	4712031- 4712547	Coding
RelE-like domain		*relE2*	SL4380	STM4450		
AbrB -like domain	*vapBC*	*vapB*	SL3012	STM3034	3216651- 3215876	Complementary
PIN-like domain		*vapC*	SL3011	STM3033		
AbrB -like domain	*vapBC*	*vapB2*	PSLT107	PSLT107	6736- 7364	Coding
PIN-like domain		*vapC2*	PSLT106	PSLT106		
-	*hok/gef*	*hok-sok*	-	-	41921- 42073	Coding
-	*ibs*	*ibsA-sibA*	-	-	3383044- 3383103	Coding
-	*ibs*	*ibsB-sibB*	-	-	2211602- 2211658	Coding
-	*ldr/fst*	*ldrA-rdlA*	-	-	3829510- 3829724	Complementary
-	*ldr/fst*	*ldrB-rdlB*	-	-	466721- 466936	Coding
-		*symER*	SL4454	STM4523	4798033- 4797630	Complementary
-	*tis*B	*tisB-istR*	-	-	4019333- 4019842	Coding

* Classification based on Makarova et al.[1] for type II modules and Fozo et al.[2] for type I modules.

† Those modules non-homologous to any other described TA module (at least toxins) were not included in any family (marked with “-“)

§ Nomenclature based on published literature, toxin-antitoxin domains and sequence homology.

Supplementary Table S2. Components of known TA modules used as PSI-BLAST queries

Protein	Toxin (T) Antitoxin (A)	Module type	Accesion no. (UNIPROT)	Organism	Toxin activity
BsrG	T	I	L8EAY0	*Bacillus subtilis*	Attacks bacterial membrane
CcdA	A	II	P62552	*Escherichia coli* plasmid F	-
CcdB	T	II	P62554	*E. coli* plasmid F	DNA gyrase inhibitor
ChpB	T	II	P33647	*E. coli* K-12	mRNA interferase
DinJ	A	II	Q47150	*E. coli K-12*	-
YafQ	T	II	Q47149	*E. coli* K-12	
GhoS	A	V	P0AF61	*E. coli* K-12	mRNA interferase
GhoT	T	V	P64646	*E. coli* K-12	mRNA interferase
HicA	T	II	P76106	*E. coli* K-12	mRNA interferase
HicB	A	II	P67697	*E. coli* K-12	-
HigA	A	II	P67701	*E. coli* K-12	-
HigB	T	II	P64578	*E. coli* K-12	mRNA interferase
HipA	T	II	P23874	*E. coli* K-12	Serine/threonine-protein kinase
HipB	A	II	P23873	*E. coli* K-12	-
Hok	T	I	P11895	*E. coli* K-12 plasmid R1	Attacks bacterial membrane
IbsA	T	I	C1P607	*E. coli* K-12	Attacks bacterial membrane
LdrD	T	I	Q6BF25	*E. coli* K-12	Attacks bacterial membrane
LsoA	T	II	O82881	*E. coli* O157:H7 plasmid pOSAK1	mRNA interferase
LsoB	A	II	Q7DKW4	*E. coli* O157:H7 plasmid pOSAK1	-
MazE	A	II	P0AE72	*E. coli* K-12	-
MazF	T	II	P0AE70	*E. coli* K-12	mRNA interferase
MqsA	A	II	Q46864	*E. coli* K-12	-
MqsR	T	II	Q46865	*E. coli* K-12	mRNA interferase
PezA	A	II	Q97QZ2	*Streptococcus pneumoniae*	
PezT	T	II	Q97QZ1	*Streptococcus pneumoniae*	UDP-N-acetylglucosamine kinase
PasT	T	II	P0AGL5	*E. coli* K-12	Inhibitor of ribosome subunit association
PasI	A	II	P52119	*E. coli* K-12	-
RelB	A	II	P0C079	*E. coli* K-12	-
RelE	T	II	P0C077	*E. coli* K-12	mRNA interferase
RnlA	T	II	P52129	*E. coli* K-12	mRNA interferase
ShoB	T	I	C1P611	*E. coli* K-12	Attacks bacterial membrane
SymE	T	I		*E. coli* K-12	mRNA interferase

Protein	Toxin (T) Antitoxin (A)	Module type	Accesion no. (UNIPROT)	Organism	Toxin activity
ToxN	T	III	B8X8Z0	*Pectobacterium atrosepticum (Erwinia carotovora subsp. atroseptica)* plasmid pECA1039	mRNA interferase
YafN	A	II	Q47156	*E. coli* K-12	-
YafO	T	II	Q47157	*E. coli* K-12	mRNA interferase
YafQ	T	II	Q47149	*E. coli* K-12	mRNA interferase
YafW	A	IV	Q47684	*E. coli* K-12	-
YeeU	A	IV	P76364	*E. coli* K-12	-
YeeV (CbtA)	T	IV	P64524	*E. coli* K-12	Inhibitor of cell division
YefM	A	II	P69346	*E. coli* K-12	-
YhaV	T	II	P64594	*E. coli* K-12	mRNA interferase
YkfI	T	IV	P77692	*E. coli* K-12	Inhibitor of cell division
YoeB	T	II	P69348	*E. coli* K-12	mRNA interferase
ε	A	II	Q57231	*Streptococcus pyogenes* plasmid pSM19035	-
ς	T	II	Q54944	*S. pyogenes* plasmid pSM19035	UDP-N-acetylglucosamine kinase

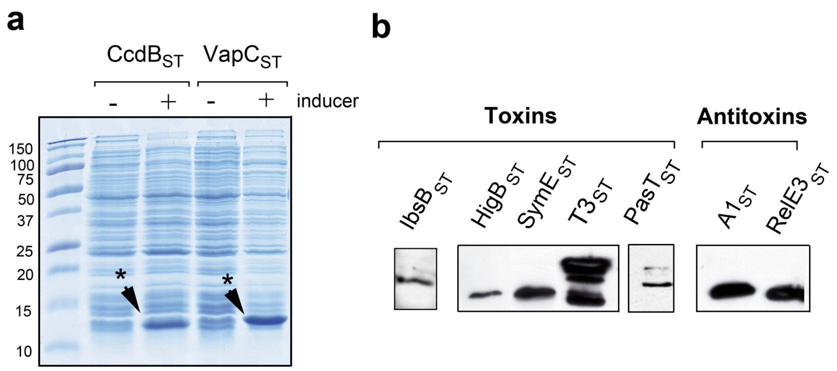

Supplementary Fig. S1: Experimental evidence for the production of toxins and antitoxins encoded by type I and type II TA modules exhibiting no activity in functional assays. (a) levels of the indicated toxins in response to inducer used to increase expression from the respective plasmids. Arrows indicate the presence of the toxin upon inducer addition. Molecular weight of standards are indicated in kDa; (b) levels of the indicated toxins and antitoxins detected by Western blotting in strains bearing expression plasmids in which 3xFLAG-tagged alleles of the respective genes were. Only the samples prepared in the presence of the inducer are shown.

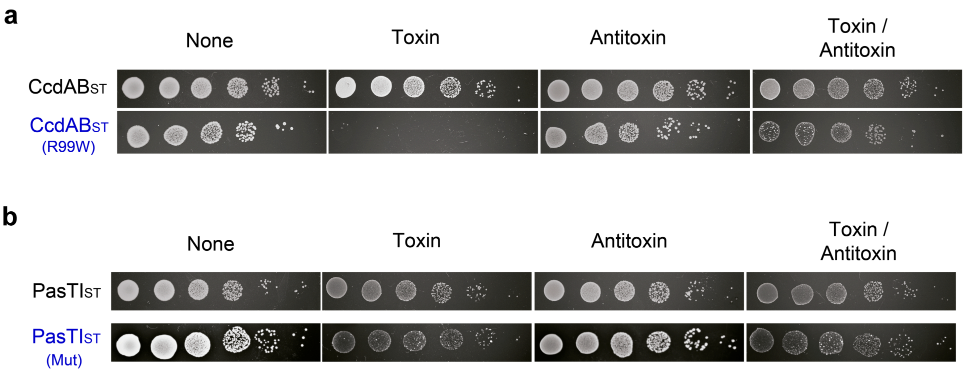

Supplementary Fig. S2. Recovery by site directed mutagenesis of anti-proliferative activity in the non-functional toxins CCdB$_{ST}$ and PasT$_{ST}$ of *S.* Typhimurium strain SL1344. (a) Inactive CcdB$_{ST}$ toxin of virulence plasmid pSLT regains full toxic activity when a key residue for gyrase-dependent toxicity is replaced by the residue present in CcdB of *E. coli* plasmid F (change R99W); (b) Inactive PasT$_{ST}$ of *S.* Typhimurium becomes partially toxic when its N-terminal region is replaced by the N-terminus of *E.coli* PasT toxin. Top figures in each panel (CcdAB$_{ST}$ and PasTI$_{ST}$) refer to the data shown for these two toxins in Figure 2 of main text.

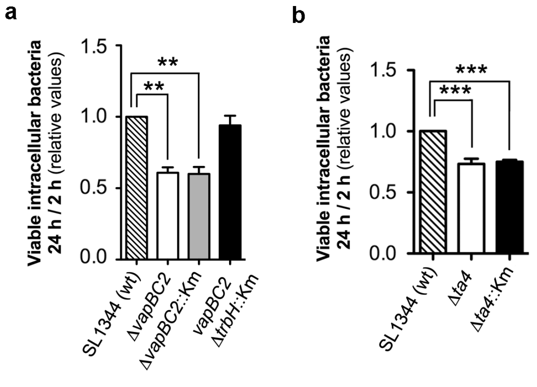

Supplementary Fig. S3. Control experiments discard polar effects due to genetic procedure involving replacement of functional TA loci. (a) Intracellular survival rates shown by isogenic strains defective in *vapBC2*$_{ST}$ carrying the Km cassette compared to the clean deletion mutant. A third mutant carrying a Km cassette in the flanking gene *trbH,* which forms part of the conjugative *tra* operon mapping in the complementary strand, was included in the assay; (b) Intracellular survival rate of the original mutant defective in *ta4*$_{ST}$, which carries a Km cassette replacing the *ta4* locus compared to the clean deletion mutant. Note that the phenotype between the kanamycin-containing strains and the clean deletion mutants is undistinguishable. Data are the means and standard deviations from three independent experiments. **, *P* = 0.001 to 0.01; ***, *P* ≤ 0.001 by one-way ANOVA with Dunnett's multiple comparison post-test.

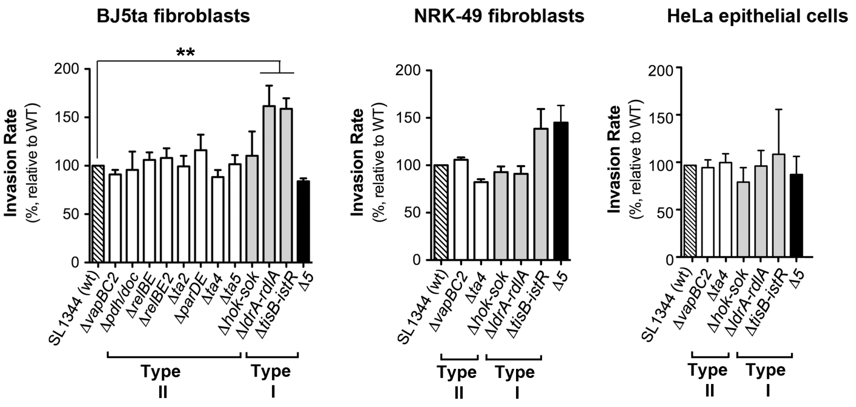

Supplementary Fig. S4. Invasion rate of two fibroblast cell lines (BJ5ta and NRK-49F) and an epithelial cell line (HeLa) displayed by *S.* Typhimurium strains lacking *bona fide* TA modules. Shown are mutants in type I (grey bars) and type II (white bars) TA modules. "Δ5" refers to the Δ*hok-sok*$_{ST}$ Δ*tisB-istR*$_{ST}$ Δ*ldrA-ldrA*$_{ST}$ Δ*ta4*$_{ST}$ Δ*vapBC2*$_{ST}$ mutant. Data are the means and standard deviations from three independent experiments. **, *P* = 0.001 to 0.01 by one-way ANOVA with Dunnett's multiple comparison post-test. Invasion rate is measured as the ratio of the cfu 2h post-infection compared to the cfu used to infect the different cell lines.

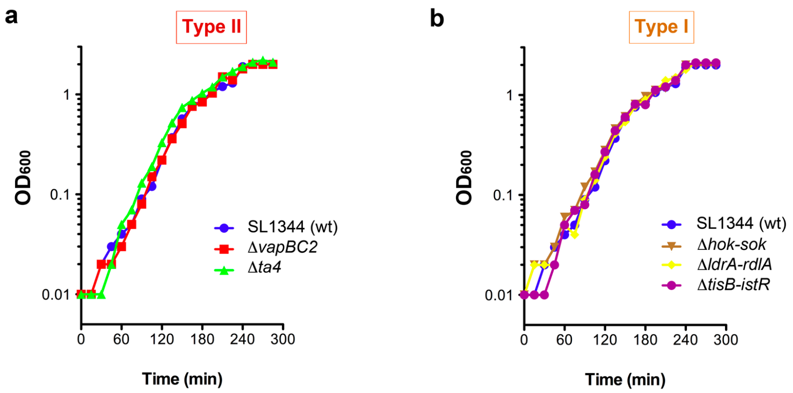

Supplementary Fig. S5*. Growth curves in LB medium of defined *S.* Typhimurium mutants lacking type I and type II TA modules required for survival inside eukaryotic cells* (see Fig.5 of main manuscript). Shown are the growth curves of parental and the indicated mutant strains in LB medium, 37°C and shaking conditions (125 rpm). (a) mutants lacking individual type II TA modules; (b) mutants lacking individual type I TA modules.

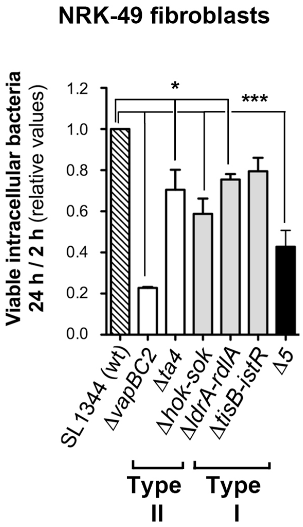

Supplementary Fig. S6*. Survival of defined *S.* Typhimurium mutants lacking type I and type II TA modules in NRK-49F rat fibroblasts.* Shown are the set of *S.* Typhimurium mutants that were tested in BJ5-ta human fibroblasts (see Fig.5 of main manuscript). Note the requirement of selected toxins of type I (grey bars) and type II (white bars) TA modules for pathogen survival. "Δ5" refers to the Δ$hok\text{-}sok_{ST}$ Δ$tisB\text{-}istR_{ST}$ Δ$ldrA\text{-}ldrA_{ST}$ Δ$ta4_{ST}$ Δ$vapBC2_{ST}$ mutant. Data are the means and standard deviations from three independent experiments. *, P = 0.1 to 0.05; ***, P = 0.001 to 0.01, by *t*-test analysis.

Supplementary Table S3. Bacterial strains and plasmids used in this study

Bacterial species / plasmid	Strain	Relevant genotype	Reference
E. coli	MG1655	F-, λ-, *rph-1*	[3]
*S.*Typhimurium	SL1344	*hisG64*, *rpsL*	[4]
	SL1344-01	$ta5_{ST}$::Km	This work
	SL1344-02	$phd\text{-}doc_{ST}$::Km	This work
	SL1344-03	$ta2_{ST}$::Km	This work
	SL1344-04	$parDE_{ST}$::Km	This work
	SL1344-05	$ta4_{ST}$::Km	This work
	SL1344-06	$\Delta ta4_{ST}$	This work
	SL1344-07	$relBE_{ST}$::Km	This work
	SL1344-08	$vapBC2_{ST}$::Km	This work
	SL1344-09	$\Delta vapBC2_{ST}$	This work
	SL1344-10	$vapBC2_{ST}$/*trbH*::Km	This work
	SL1344-11	$hok\text{-}sok_{ST}$::Km	This work
	SL1344-12	$ldrA\text{-}rdlA_{ST}$::Km	This work
	SL1344-13	$tisB\text{-}istR_{ST}$::Km	This work
	SL1344-14	$t2_{ST}$-3xFLAG	This work
	SL1344-15	$t4_{ST}$-3xFLAG	This work
	SL1344-16	$t5_{ST}$-3xFLAG	This work
	SL1344-17	$vapC2_{ST}$-3xFLAG	This work
	SL1344-21	$\Delta ta4_{ST}$/Δ*vapBC2/Δhok-sok/ΔldrA-rdlA* *ΔtisB-istR*::Km	This work
Plasmids	pACYC184	*cat, tet*	[5]
	pFUS2	*aph*	[6]
	pAC-P_{lac}	pACYC184 derived plasmid. P_{lac} promoter,	This work
	pFUS-P_{BAD}	pBR322 derived plasmid. P_{BAD} promoter,	This work
	pKD267	*kanamycin-parE* cassette	[7]
	pKD46	λRed recombinase	[8]
	pSUB11	kanamycin-*3xFLAG* cassette	[9]
	pCP20	*bla,* induction of flippase synthesis by temperature	[10]

Supplementary Table S4. Oligonucleotides used as primers in this study

Name	Primer sequence (5' – 3')	Used for
T1-zra	GCC*GACGTC*ATGAAAAGATTACGCAGTAAAATG	Cloning toxin t1. ZraI site underlined
T1-spe	GCC*ACTAGT*TTAGTCCGCCTCATTGATACCTAAACG	Cloning toxin t1. SpeI site underlined
A1-zra	GCC*GACGTC*ATGTTCAAGGAACGGATGACGCCAGAAG	Cloning antitoxin a1. ZraI site underlined
A1-spe	GCC*ACTAGT*TTATTTGCTATCGCGTATCCCTAAGCGTTG	Cloning antitoxin a1. SpeI site underlined
T2-zra	GCC*GACGTC*ATGATGTTTACAGACTGGCATGAG	Cloning t2 toxin. ZraI site underlined
T2-spe	GCG*ACTAGT*GTCATAACCTACCAGATGCAGATAAAG	Cloning t2 toxin. SpeI site underlined
A2-zra	GCC*GACGTC*ATGAAAACCATGCCTCAGATAGC	Cloning a2 antitoxin. ZraI site underlined
A2-spe	GCG*ACTAGT*CCAGTCTGTAAACATCATTTCTTC	Cloning a2 antitoxin. SpeI site underlined
ParE-zra	GCC*GACGTC*ATGGTAAAATTAACGCCAAAG	Cloning *parE* toxin. ZraI site underlined
ParE-spe	GCC*ACTAGT*CTATAACCAATTTACATGCTTAC	Cloning *parE* toxin. SpeI site underlined
ParD-zra	GCC*GACGTC*ATGACCGTTGATCTTGGCGATGAA	Cloning *parD* antitoxin. ZraI site underlined
ParD-spe	GCC*ACTAGT*TCACTGGCCTTTGGCGTTAATTTTACC	Cloning *parD* antitoxin. SpeI site underlined
T3-zra	GCC*GACGTC*ATGAGCGATAAATTTGGCGAAGG	Cloning *t3* toxin. ZraI site underlined
T3-spe	GCC*ACTAGT*TTACTCAGTTTCGCGAGCTTCGCTTATC	Cloning *t3* toxin. SpeI site underlined
A3-zra	GCC*GACGTC*GTGAAGAAACTTACCGATAAACAAAAGTC	Cloning *a3* antitoxin. ZraI site underlined
A3-spe	GCC*ACTAGT*ATTTATCGCTCATAGTGCCTCCGAAG	Cloning *a3*antitoxin. SpeI site underlined
T4-zra	GGA*GACGTC*GTGGGACGTGTAACAGCACCAGAACCTTTG	Cloning *t4* toxin. ZraI site underlined
T4-spe	GGC*ACTAGT*CTATTGAGGGAGCCTAAGGAACAATGTTC	Cloning *t4* toxin. SpeI site underlined
A4-zra	GGC*GACGTC*ATGCTATACAAGGGGTGTCTCATGAAATC	Cloning *a4* antitoxin. ZraI site underlined
A4-spe	GGC*ACTAGT*TTACACGTCCCACTGAGGTTTC	Cloning *a4* antitoxin. SpeI site underlined
RelE3-zra	GGC*GACGTC*ATGCGAACCTTCAAAACCAGGTG	Cloning *relE3* toxin. ZraI site underlined
RelE3.2-zra	GCG*GACGTC*GTGTATCACCTGGTGCTATAC	Cloning *relE3* N-extended toxin. ZraI site underlined

RelE3-spe	GGC***ACTAGT***TAGTTTTTGCTGACATGGCGCACCTCTAC	Cloning *relE3*toxin. SpeI site underlined
RelB3-zra	GGC***GACGTC***ATGTCAGCAAAAACTAAATTC	Cloning *relB3*antitoxin. ZraI site underlined
RelB3-spe	GCC***ACTAGT***CATACCAGCACCTTTAGC	Cloning *relB3*antitoxin. SpeI site underlined
SehA-zra	GCT***GACGTC***GTGCATGTTATCAGCCGAAAACC	Cloning *sehA* toxin. ZraI site underlined
SehA -spe	GCC***ACTAGT***TCATTCTTTATTACCCCGATAGTATGC	Cloning *sehA* toxin. SpeI site underlined
SehB -zra	GGC***GACGTC***ATGGATGCAACCAGCGCAAAAAAGATCGTTG	Cloning *sehB* antitoxin. ZraI site underlined
SehB -spe	GGC***ACTAGT***CTACTCGATGAAGGCATCTGCTGGTAGTTTG	Cloning *sehB* antitoxin. SpeI site underlined
SehD -zra	GCC***GACGTC***ATGCAATTTATAGAAACGGAAC	Cloning *sehD* toxin. ZraI site underlined
SehD-spe	GCC***ACTAGT***CTACCACCTCTCATTCAGCATAC	Cloning *sehD* toxin. SpeI site underlined
SehC -zra	GCC***GACGTC***ATGGATAAAGTGTTATTTGAGCGATTAAC	Cloning *sehC* antitoxin. ZraI site underlined
SehC-spe	GCC***ACTAGT***TCAATAACGCAATGCTTGTATAACGTTTC	Cloning *sehC* antitoxin. SpeI site underlined
T5-zra	GCC***GACGTC***ATGATCTCCACCCCTGAG	Cloning *t5* toxin. ZraI site underlined
T5-spe	GCC***ACTAGT***GATGCGTTCTGGAGTTTAAC	Cloning *t5* toxin. SpeI site underlined
A5-zra	GCC***GACGTC***ATGCCAGCCGCAAACAGTATG	Cloning *a5* antitoxin. ZraI site underlined
A5-spe	GCC***ACTAGT***CTCAGGGGTGGAGATCATTTTTCC	Cloning *a5* antitoxin. SpeI site underlined
ShpA-zra	GCC***GACGTC***ATGCCGATGGAGTTTGAATGGGATGC	Cloning *shpA* toxin. ZraI site underlined
ShpA-spe	GCC***ACTAGT***TTAACCATGCTCATAACGATTCCTCTC	Cloning *shpA* toxin. SpeI site underlined
ShpB-zra	GCC***GACGTC***ATGAGCATGGTTAAACATAAAC	Cloning *shpB* antitoxin. ZraI site underlined
ShpB-spe	GCC***ACTAGT***CGGGCTATTTCTTATTTTGCTC	Cloning *shpB* antitoxin. SpeI site underlined
RelE4-zra	GCC***GACGTC***ATGATGGAGATATTCTGGACCATG	Cloning *relE4* toxin. ZraI site underlined
RelE4-spe	GCC***ACTAGT***CTATGGCCACTTCTGTGCAGTATG	Cloning *relE4* toxin. SpeI site underlined
RelB4-spe	GCC***ACTAGT***ATGGCACAGGTTAATATGAGTTTAAG	Cloning *relB4* antitoxin. SpeI site underlined
RelB4-spe2	GCC***ACTAGT***TCATTTACCGGCAACCTTCC	Cloning *relB4* antitoxin. SpeI site underlined
CcdB-zra	GCC***GACGTC***ATGCAGTTTAAGGTTTACAC	Cloning *ccdB* toxin. ZraI site underlined
CcdB-spe	GCC***ACTAGT***TCAGATCCCCCGGAACATC	Cloning *ccdB* toxin. SpeI site underlined

CcdB-mut-5	CCATTAACCTGATGTTC**T**GGGGGATCTGAACTAG	Introduction mutation R99W in *ccdB*. Nucleotide changed is underlined.
CcdB-mut-3	CTAGTTCAGATCCCCC**A**GAACATCAGGTTAATGG	Introduction mutation R99W in *ccdB*. Nucleotide changed is underlined.
CcdA-zra	GCC***GACGTC***ATGAAGCAGCGAATTACAGTGACAG	Cloning *ccdA* antitoxin. ZraI site underlined
CcdA-spe	GCC***ACTAGT***TCACCAGTTCCTGTTGTCGTCAGCAAAC	Cloning *ccdA* antitoxin. SpeI site underlined
DinJ-zra	GCC***GACGTC***ATGGCTGCAAATGCGCTTG	Cloning *dinJ* antitoxin. ZraI site underlined
DinJ-spe	GCC***ACTAGT***TCAGATCCCTAACTGGTCAAACAAATC	Cloning *dinJ* antitoxin. SpeI site underlined
YafQ-zra	GCC***GACGTC***ATGGGGCAAAGGGAAATTGAATATTC	Cloning *yafQ* toxin. ZraI site underlined
YafQ-spe	GCC***ACTAGT***TTAAAATAAATCGGCATGCGTTCCTGTTCGTTC	Cloning *yafQ* toxin. SpeI site underlined
IbsA-zra	GCC***GACGTC***ATGATGCACCAGGTCATCATAC	Cloning *ibsA* toxin. ZraI site underlined
IbsA-spe	GCC***ACTAGT***CCTCTGATTGTCTGTTAGTAAG	Cloning *ibsA* toxin. SpeI site underlined
IbsB-zra	GCC***GACGTC***ATGTGGCTTAACGTCAGGAGTGAGGGTAAG	Cloning *ibsB* toxin. ZraI site underlined
IbsB-spe	GCC***ACTAGT***TGATTAGCCTACCAGCTTACTAAGACAC	Cloning *ibsB* toxin. SpeI site underlined
HigB-zra	GCG***GACGTC***GTGGGATCTTCTCTGGAGGATC	Cloning *higB* toxin. ZraI site underlined
HigB-spe	GCC***ACTAGT***TCACAAGCTGTTTCTCCGTTGTTGC	Cloning *higB* toxin. SpeI site underlined
HigA-zra	GCG***GACGTC***GTGATTGCCAAAACTGATAG	Cloning *higA* antitoxin. ZraI site underlined
HigA-spe	GCC***ACTAGT***TTACGGCCCTACCATTACC	Cloning *higA* antitoxin. SpeI site underlined
Hok-zra	GCC***GACGTC***ATGCCACAGCGAACGTTTTTAATG	Cloning *hok* toxin. ZraI site underlined
Hok-spe	GCC***ACTAGT***TTAACGTTTAACTTCGTAGGCTAAC	Cloning *hok* toxin. SpeI site underlined
Doc-zra	GCC***GACGTC***ATGACCCTACAACTTATCTCAG	Cloning *doc* toxin. ZraI site underlined
Doc-spe	GTA***ACTAGT***CCGGGATTAACGTCTCAGG	Cloning *doc* toxin. SpeI site underlined
Phd-zra	GGCC***GACGTC***ATGTTTATGCGTACGGTTAACTATAGCG	Cloning *phd* antitoxin. ZraI site underlined
Phd-spe	GGT***ACTAGT***GGTCATTTATCCGCCAGCTCCCTGAG	Cloning *phd* antitoxin. SpeI site underlined
LdrA-zra	GCC***GACGTC***ATGACGCTTACGCAGTTGGGCGTGGTCTTCTG	Cloning *ldrA* toxin. ZraI site underlined
LdrA-spe	GCC***ACTAGT***TACTTCCTGTCACGTAGCCAGTTGACGATC	Cloning *ldrA* toxin. SpeI site underlined

LdrB-zra	GCC***GACGTC***ATGACGCTCACAGAGTTGAGCATTACTATC	Cloning *ldrB* toxin. ZraI site underlined
LdrB-spe	GCC***ACTAGT***CGCCCACTTCACATTACTTCCG	Cloning *ldrB* toxin. SpeI site underlined
PasT-zra	GCG***GACGTC***GTGGTATTATTTACACGATTTATGTTG	Cloning *pasT* toxin. ZraI site underlined
PasT-spe	GCC***ACTAGT***TTATCCGGCACGGTAAACCTCTTTG	Cloning *pasT* toxin. SpeI site underlined
PasT-mut-zra	***GACGTC***ATGATATTATTTGTAGGATTTCTGTTGATGGGAATTGCTATGC	Introduction N-terminal mutation in *pasT*.
PasI-zra	GCG***GACGTC***GTGCCGGATAAACTTGTGGTTG	Cloning *pasI* antitoxin. ZraI site underlined
PasI-spe	GCG***ACTAGT***AAAAGAGGCTAATTATCTACCAG	Cloning *pasI* toxin. SpeI site underlined
RelE-zra	GCC***GACGTC***ATGACTTATAAGCTGGCATTTAACG	Cloning *relE* toxin. ZraI site underlined
RelE-spe	GCC***ACTAGT***TTAGCTTCTGTGTCGTGTCATTTTG	Cloning *relE* toxin. SpeI site underlined
RelB-zra	GCC***GACGTC***ATGGCATTTCAAATTTTAACGACTAC	Cloning *relB* antitoxin. ZraI site underlined
RelB-spe	GCC***ACTAGT***TTATAAGTCATCAATGTTGACCTCAATG	Cloning *relB* antitoxin. SpeI site underlined
RelE2-zra	GCC***GACGTC***ATGACTTATGAACTGGAATTCG	Cloning *relE2* toxin. ZraI site underlined
RelE2-spe	GCC***ACTAGT***ATCGCTAAAGCCGTTTGTTTG	Cloning *relE2* toxin. SpeI site underlined
RelB2-zra	GCC***GACGTC***ATGGCCACGCTGAACGTCCGTCTGGATGACAAACTC	Cloning *relB2* antitoxin. ZraI site underlined
RelB2-spe	GCC***ACTAGT***TCATAAGTCATCCAGACTAACCTTGATTC	Cloning *relB2* antitoxin. SpeI site underlined
SymE-zra	GCC***GACGTC***ATGACTACCGTCCATTCTATTG	Cloning *symE* toxin. ZraI site underlined
SymE-spe	GCC***ACTAGT***ATTTTATGCGTTTACTTAGAACCTG	Cloning *symE* toxin. SpeI site underlined
TisB-zra	GCC***GACGTC***ATGAGCGTAGTGGATATCACCATTC	Cloning *tisB* toxin. ZraI site underlined
TisB-spe	GCG***ACTAGT***GGCTTGAATCTGAATTACTTAAGG	Cloning *tisB* toxin. SpeI site underlined
VapC-zra	GCC***GACGTC***ATGCTGAAATTCATGCTTGATAC	Cloning *vapC* toxin. ZraI site underlined
VapC-spe	GCC***ACTAGT***TTAGCACCAGTCTTCGATTCGGATACC	Cloning *vapC* toxin. SpeI site underlined
VapB-zra	GCC***GACGTC***ATGTATTCAGAAATGCCGGAGTCGGAC	Cloning *vapB* antitoxin. ZraI site underlined
VapB-spe	GCC***ACTAGT***TCAAAATCCTTCCCGTTCCTGTACTG	Cloning *vapB* antitoxin. SpeI site underlined
VapC2-zra	GCC***GACGTC***ATGCTGAAGTTTATGCTGGATACTAAC	Cloning *vapC2* toxin. ZraI site underlined
VapC2-spe	GCC***ACTAGT***TCAGCTCCAGTCCTCAGTTCTCAG	Cloning *vapC2* toxin. SpeI site underlined
VapB2-zra	GCC***GACGTC***ATGATGGAAACCAGCGTATTTCTC	Cloning *vapB2* antitoxin. ZraI site underlined

VapB2-spe	GCC***ACTAGT***TCAGAATGATTCCCGTTCCTG	Cloning *vapB2* antitoxin. SpeI site underlined
Fw-del-Phd-Doc	GCGAACGAAGCGGTGCGCAGCGCCTATCTGGGCGGTTAGACTGTGCCAG GTCTCTACGCCGGACGCATCGTG	Amplification *km-parE* cassette from pKD267 to delete *phd-doc*.
Rv-del-Phd-Doc	CGGCGCACATGCCGTAACCCAACCAGGCGGAGCGTCGCCGGGATTAACG TCACTGATCAGTGATAAGCTGTC	Amplification *km-parE* cassette from pKD267 to delete *phd-doc.*
Fw-del-TA2	GCCTGGACTATATCAGCCTCATATGTACGCCTTGAAAGCGTACAGATATG TCTCTACGCCGGACGCATCGTG	Amplification *km-parE* cassette from pKD267 to delete *ta2*.
Rv-del-TA2	CCTGTATCCTTATCGTTGGCCGATTCTGAATTATTCAACAAAGCCTCATGT TGACTGATCAGTGATAAGCTGTC	Amplification *km-parE* cassette from pKD267 to delete *ta2.*
Fw-del-ParDE	GGCTCAGAATACGTATCACGATAACTTCCGTAACTGATTGTATAAAATAGT CTCTACGCCGGACGCATCGTG	Amplification *km-parE* cassette from pKD267 to delete *parDE*.
Rv-del- ParDE	CCGGTGTACTCTTATGTAAGATTTATACTTACAGTGGAGGCTGTTATGGCC ACTGATCAGTGATAAGCTGTC	Amplification *km-parE* cassette from pKD267 to delete *parDE.*
Fw-del-TA4	GACATTCTATCAAATTATCGCTTATAGCGATTTGAACATAACAGTCTTTGTC TCTACGCCGGACGCATCGTG	Amplification *km-parE* cassette from pKD267 to delete *ta4*.
Rv-del-TA4	CCGCTTCTGGCACAAAGCGAACATCAGTATTCAAAAAATGAAAAACGACG AACTGATCAGTGATAAGCTGTC	Amplification *km-parE* cassette from pKD267 to delete *ta4*.
TA4-5.1	CAGTAAAGGCGCTGTCATCGTACAAAAAG	Amplification *ta4* upstream region
TA4-3.1	GCCCTCGAGCAAAGACTGTTATGTTCAAATCG	Amplification *ta4* upstream region
TA4-5.2	GCCCTCGAGTCGTCGTTTTTCATTTTTTGAATACTG	Amplification *ta4* downstream region
TA4-3.2	GCGAGGAACGGACGCCCTATTTCAC	Amplification *ta4* downstream region
Fw-del-TA5	GACATCTGCGTTACGCGTGTTGTATGCTTAATATCCACAGAAGTACAAGA GTCTCTACGCCGGACGCATCGTG	Amplification *km-parE* cassette from pKD267 to delete *ta5*.
Rv-del-TA5	TTAAACACTCTCCACCAAATCCCCCAACGTCACCATCAACATCATCGGATC ACTGATCAGTGATAAGCTGTC	Amplification *km-parE* cassette from pKD267 to delete *ta5*.

Fw-del-RelBE	GAGCCTGTTTGTCGTATCGTTTGACGGGAAAGAGAGCATTTTCTGAGTGA TCTCTACGCCGGACGCATCGTG	Amplification *km-parE* cassette from pKD267 to delete *relBE.*
Rv-del-RelBE	CCGGTTTCAGTGGTCATAAGTTAGGTTCTGGACATCTGAAATTAGCCTTG ACTGATCAGTGATAAGCTGTC	Amplification *km-parE* cassette from pKD267 to delete *relBE.*
Fw-del-VapBC2	TAACCGGCGGCACTTCCCGCCGACCGGACCTGCGCAATACTCATCATAA ATCTCTACGCCGGACGCATCGTG	Amplification *km-parE* cassette from pKD267 to delete *vapBC2.*
Rv-del-VapBC2	CTGATACCGCCATCAGAAATCATCTCCCGGCTCCGGTTCATCCTTTCCGT ACTGATCAGTGATAAGCTGTC	Amplification *km-parE* cassette from pKD267 to delete *vapBC2.*
VapBC2-5.1	CCGTTTTGCCCACCTTATCGCTGCTC	Amplification *vapBC2* upstream region
VapBC2-3.1	GGAGATGATTTCTGATGGCGGTATCAGTTTATGATGAGTATTGCGCAGG TCCGGTC	Amplification *vapBC2* upstream region
VapBC2-5.2	GGACCTGCGCAATACTCATCATAAACTGATACCGCCATCAGAAATCATCT C	Amplification *vapBC2* downstream region
VapBC2-3.2	GCTGGCCCTGAAATACCAGGAAC	Amplification *vapBC2* downstream region
Fw-del-TisB	CGGGATTGATGATCACGCATTTCACAATGCCGGAAAACAAAAAACCTCG CTCTCTACGCCGGACGCATCGTG	Amplification *km-parE* cassette from pKD267 to delete *tisB-istR.*
Rv-del-TisB	CCCTTCGGTGCGGCTTGAATCTGAATTACTTAAGGTATTTCAGAACAGCA ACTGATCAGTGATAAGCTGTC	Amplification *km-parE* cassette from pKD267 to delete *tisB-istR.*
Fw-del-LdrA	ACGCGTTACTTCCTGTCACGTAGCCAGTTGACGATCACACTGGCGATAA TTCTCTACGCCGGACGCATCGTG	Amplification *km-parE* cassette from pKD267 to delete *ldrA-rdlA.*
Rv-del- LdrA	CAGCAAGCCGGGTTTACCCGGTGAGGCGCAATGTTGCGGGGGTTTGAT CCACTGATCAGTGATAAGCTGTC	Amplification *km-parE* cassette from pKD267 to delete *ldrA-rdlA.*
LdrA-5.1	CCGGGCGTAAGGTGATGGTAG	Amplification *ldrA-rdlA* upstream region
LdrA-3.1	AATGTTGCGGGGGTTTGATCCATTATCGCCAGTGTGATCGTCAACT	Amplification *ldrA-rdlA* upstream region
LdrA-5.2	AGTTGACGATCACACTGGCGATAATGGATCAAACCCCCGCAACATT	Amplification *ldrA-rdlA* downstream region
LdrA-3.2	CCAGGCTATAGTGCCTTGATACC	Amplification *ldrA-rdlA* downstream region

Fw-del-Hok	TAATGCCTAGACAACATTATAGTAGCCCGATAACCGCCGTAAGGCAATGT CTCTACGCCGGACGCATCGTG	Amplification *km-parE* cassette from pKD267 to delete *hok-sok.*
Rv-del-Hok	AAACAATACCGGATACGCTTCATTGAAGAAGAAAGGGCGCAATGAGGTC AACTGATCAGTGATAAGCTGTC	Amplification *km-parE* cassette from pKD267 to delete *hok-sok.*
Hok-5.1	GCTTATGTTGGCGGCGTTGTTAC	Amplification *hok-sok* upstream region
Hok-3.1	GAAGAAAGGGCGCAATGAGGTCACATTGCCTTACGGCGGTTATCG	Amplification *hok-sok* upstream region
Hok-5.2	CGATAACCGCCGTAAGGCAATGTGACCTCATTGCGCCCTTTCTTC	Amplification *hok-sok* downstream region
Hok-3.2	GCTGACATGCCGAGTATACAC	Amplification *hok-sok* downstream region
Fw-tag-VapC2	ACACCCGGGAGTTTGAACGTGTGGGTGGACTGAGAACTGAGGACTGGA GCGACTACAAAGACCATGACGGTG	Amplification *km-3xFLAG* cassette from pSUB11 to tag VapC2.
Rv-tag-VapC2	CGGAAAGGATGAACCGGAGCCGGGAGATGATTTCTGATGGCGGTATCA GCATATGAATATCCTCCTTAGTTCC	Amplification *km-3xFLAG* cassette from pSUB11 to tag VapC2.
Fw-tag-T2	GTTGCTGTCGTTTAAAACGCTTTATGCTGCTTTATCTGCATCTGGTAGGT TAGACTACAAAGACCATGACGGTG	Amplification *km-3xFLAG* cassette from pSUB11 to tag T2.
Rv-tag-T2	GTATCCTTATCGTTGGCCGATTCTGAATTATTCAACAAAGCCTCATGTTG TCACATATGAATATCCTCCTTAGTTCC	Amplification *km-3xFLAG* cassette from pSUB11 to tag T2.
Fw-tag-T4	TCAAATCATCACAAACTCAGCAGCGAACATTGTTCCTTAGGCTCCCTCAA GACTACAAAGACCATGACGGTG	Amplification *km-3xFLAG* cassette from pSUB11 to tag T4.
Rv-tag-T4	TTCTATCAAATTATCGCTTATAGCGATTTGAACATAACAGTCTTTGCATTC TACATATGAATATCCTCCTTAGTTCC	Amplification *km-3xFLAG* cassette from pSUB11 to tag T4.
Fw-tag-T5	GGATCCGATGATGTTGATGGTGACGTTGGGGGATTTGGTGGAGAGTGTT GACTACAAAGACCATGACGGTG	Amplification *km-3xFLAG* cassette from pSUB11 to tag T5.
Rv-tag-T5	CACAGCGAATTCCGGCCATCAGAAGCTAAGGGATGCGTTCTGGAGTTTA CATATGAATATCCTCCTTAGTTCC	Amplification *km-3xFLAG* cassette from pSUB11 to tag T5.
pFUS-sec	CTGTATCAGGCTGAAAATCTTCTCTC	Sequencing of cloned toxin/antitoxin gene
pAC-sec	CATAATGGGGAAGGCCATCCAG	Sequencing of cloned toxin/antitoxin gene

hok Fw	GTCTGGATGGTGAGGGATTCG	RT-qPCR
hok Rv	CAAGCACTGTGTTTCCCTGC	RT-qPCR
ibsA Fw	GCACCAGGTCATCATAC	RT-qPCR
ibsA Rv	GCTGCGAAACTTATC	RT-qPCR
ldrA Fw	GGGCGTGGTCTTCTGGCACG	RT-qPCR
ldrA Rv	TTACTTCCTGTCACGTAGCC	RT-qPCR
ldrB Fw	TTGAGCATTACTATCTGGCACGAT	RT-qPCR
ldrB Rv	AAAAACAGACCTGTCGCAATCC	RT-qPCR
tisB Fw	GCGTAGTGGATATCACCATT	RT-qPCR
tisB Rv	TTAAGGTATTTCAGAACAGC	RT-qPCR

References

1. Makarova KS, Wolf YI, Koonin EV. Comprehensive comparative-genomic analysis of Type 2 toxin-antitoxin systems and related mobile stress response systems in prokaryotes. *Biology direct* **4**, 19 (2009).

2. Fozo EM, Makarova KS, Shabalina SA, Yutin N, Koonin EV, Storz G. Abundance of type I toxin-antitoxin systems in bacteria: searches for new candidates and discovery of novel families. *Nucleic Acids Res.* **38**, 3743-3759 (2010).

3. Blattner FR*, et al.* The complete genome sequence of *Escherichia coli* K-12. *Science* **277**, 1453-1462 (1997).

4. Hoiseth SK, Stocker BA. Aromatic-dependent *Salmonella typhimurium* are non-virulent and effective as live vaccines. *Nature* **291**, 238-239 (1981).

5. Chang AC, Cohen SN. Construction and characterization of amplifiable multicopy DNA cloning vehicles derived from the P15A cryptic miniplasmid. *J. Bacteriol.* **134**, 1141-1156 (1978).

6. Lemonnier M, Lane D. Expression of the second lysine decarboxylase gene of *Escherichia coli*. *Microbiology* **144**, 10 (1998).

7. Maisonneuve E, Shakespeare LJ, Jorgensen MG, Gerdes K. Bacterial persistence by RNA endonucleases. *Proc Natl Acad Sci U S A* **108**, 13206-13211 (2011).

8. Datsenko KA, Wanner BL. One-step inactivation of chromosomal genes in *Escherichia coli* K-12 using PCR products. *Proc Natl Acad Sci U S A* **97**, 6640-6645 (2000).

9. Uzzau S, Figueroa-Bossi N, Rubino S, Bossi L. Epitope tagging of chromosomal genes in *Salmonella*. *Proc. Natl. Acad. Sci. U S A* **98**, 15264-15269 (2001).

10. Cherepanov PP, Wackernagel W. Gene disruption in *Escherichia coli:* TcR and KmR cassettes with the option of Flp-catalyzed excision of the antibiotic-resistance determinant. *Gene* **158**, 9-14 (1995).
